# STEAP2 promotes hepatocellular carcinoma progression via increased copper levels and stress-activated MAP kinase activity

**DOI:** 10.1038/s41598-024-63368-2

**Published:** 2024-06-03

**Authors:** Carla Zeballos Torrez, Acarizia Easley, Hakim Bouamar, Guixi Zheng, Xiang Gu, Junhua Yang, Yu-Chiao Chiu, Yidong Chen, Glenn A. Halff, Francisco G. Cigarroa, Lu-Zhe Sun

**Affiliations:** 1 Department of Cell Systems and Anatomy, University of Texas Health Science Center at San Antonio, San Antonio, TX USA; 2 Department of Population Health Sciences, University of Texas Health Science Center at San Antonio, San Antonio, TX USA; 3 Greehey Children’s Cancer Research Institute, University of Texas Health Science Center at San Antonio, San Antonio, TX USA; 4 Transplant Center, University of Texas Health Science Center at San Antonio, San Antonio, TX USA

**Keywords:** Hepatocellular carcinoma, RNA sequencing

## Abstract

Six Transmembrane Epithelial Antigen of Prostate 2 (STEAP2) belongs to a family of metalloreductases, which indirectly aid in uptake of iron and copper ions. Its role in hepatocellular carcinoma (HCC) remains to be characterized. Here, we report that STEAP2 expression was upregulated in HCC tumors compared with paired adjacent non-tumor tissues by RNA sequencing, RT-qPCR, Western blotting, and immunostaining. Public HCC datasets demonstrated upregulated STEAP2 expression in HCC and positive association with tumor grade. Transient and stable knockdown (KD) of STEAP2 in HCC cell lines abrogated their malignant phenotypes in *vitro* and in vivo, while STEAP2 overexpression showed opposite effects. STEAP2 KD in HCC cells led to significant alteration of genes associated with extracellular matrix organization, cell adhesion/chemotaxis, negative enrichment of an invasiveness signature gene set, and inhibition of cell migration/invasion. STEAP2 KD reduced intracellular copper levels and activation of stress-activated MAP kinases including p38 and JNK. Treatment with copper rescued the reduced HCC cell migration due to STEAP2 KD and activated p38 and JNK. Furthermore, treatment with p38 or JNK inhibitors significantly inhibited copper-mediated cell migration. Thus, STEAP2 plays a malignant-promoting role in HCC cells by driving migration/invasion via increased copper levels and MAP kinase activities. Our study uncovered a novel molecular mechanism contributing to HCC malignancy and a potential therapeutic target for HCC treatment.

## Introduction

Hepatocellular carcinoma (HCC) is the most common type of liver cancer in adults and the fourth most common cause of cancer-related deaths worldwide^1,2^. HCC outcomes are poor, present with limited therapeutic options, and is the fastest-rising cause of cancer-related death in the United States (US)^3^, with incidence rates almost tripling in the US over the past twenty years^4^. The 5-year survival rate of 18% makes liver cancer the second most lethal tumor^5^. Liver cancer disproportionately affects Latinos, especially those in South Texas^6^, as South Texas Latinos have the highest incidence rate in comparison to non-Latino Whites (NLW), with a rate ratio of 3.6 among Latino males compared to NLW counterparts^7,8^. This trend has been attributed to increased incidence of diabetes and obesity, environmental, cultural, and possibly genetic alterations^7^. Further investigations are warranted to determine the cause of the higher incidence rates of HCC in the US, especially among South Texas Latinos as this unique population with increased incidence rates of HCC will provide insights, such as novel molecular markers that can help in early diagnosis and risk assessment.

The six-transmembrane epithelial antigen of the prostate (STEAP) family contains four homolog proteins (STEAP1, -2, -3, and -4), which share a characteristic transmembrane region that is flanked by intracellular amino- and carboxy-terminal domains^9,10^. The carboxy-terminal have heme-binding capabilities and are involved in electron transfer chains; the N-terminal domain is highly homologous with bacterial and archaeal metalloreductases F420:NADPH-oxidoreductase (FNO) and human NADPH-oxidoreductase (NOS)^9–13^. This FNO-like domain is predicted to enable STEAPs to bind flavins as electron donors for their oxidoreductase activity^11,14^. The N-terminal domain works in conjunction with the C-terminal transmembrane domain to carry out cell surface ferric and cupric reductase activity in STEAP 2–4^11,14^. STEAP 2–4 were demonstrated to function as metalloreductases in vitro^11,13–16^. STEAP1 and STEAP2 are highly overexpressed in numerous types of human cancers, such as prostate, bladder, pancreas, ovary, testis, breast, cervix and Ewing sarcoma^9,17,18^; however, their physiological roles in normal and cancer cells are not well understood^15,19^. Evidence suggests that STEAP2 is specifically overexpressed in invasive prostate cancer, therefore promoting proliferation, migration, and invasion^15,20^. In contrast, Yang et. al. demonstrates that STEAP2 is downregulated in breast cancer tissues and acts as an anti-oncogene in breast cancer development by suppressing EMT and blocking PI3K/AKT signaling^21^. The opposing roles of STEAP2 in prostate cancer and breast cancer highlight the need for further studies on STEAP2 in various types of cancers. In a recent study utilizing The Cancer Genome Atlas HCC data, Fu et al. demonstrates increased expression of STEAP1 and STEAP2 and decreased expression of STEAP3 and STEAP4 in HCC tumors compared to adjacent non-tumor tissue^22^. STEAP1 expression was associated with better overall survival (*p* = 0.016) while STEAP2 was not statistically significantly associated with overall survival^22^. The authors established a risk score model, which included STEAP1 and STEAP4 genes. While Fu et. al. found that STEAP2 was not a significant indicator of predicting prognosis, the lack of mechanistic insights to how STEAP2 may regulate HCC progression as a metalloreductase warrant further studies. It is well established that risk factors for HCC include elevated levels of copper and iron, which are associated with Wilson’s disease and hereditary hemochromatosis, respectively^4^. Coopper levels are elevated in serum and tumor tissue in cancer patients^23–25^, facilitating cancer growth, angiogenesis, and metastasis^26^. Meanwhile, abnormal iron uptake is observed in various types of cancers^27^ and hepatic iron overload is observed in patients with HCC^28,29^. Given these findings, a link between STEAP2, copper levels, and invasiveness may play an important role in HCC.

Copper is an essential trace element that functions as a catalytic co-factor in several enzymes due to its potent redox activity^25^. Copper can readily change between oxidized (Cu^II^) and reduced (Cu^I^) states in biological medium; this characteristic makes copper a critical co-factor in many biological processes and a key modulator of cell signal transduction pathways^25,30,31^. These pathways are involved in complex molecular interactions that drive cellular mechanisms and are associated with the interplay of key enzymes including kinases and phosphatases. Such kinases and phosphatases include those in the MAPK signaling cascade; components of the MAPK signal transduction pathways, such as c-Jun N-terminal kinase (JNK) and p38, have been reported to be activated by copper^30^. JNK and p38 MAPKs are fundamental for cellular processes such as proliferation, differentiation, apoptosis, migration, and inflammation^32^. Accumulating evidence suggests that the JNK and p38 pathway are involved in the regulation of cell migration^33–35^. The signaling molecules that activate JNK, such as MEK kinase 1, an upstream kinase in the JNK pathway, are essential for cell migration^36^. Inhibition of JNK by either the chemical inhibitor SP600125, the dominant-negative mutant JNK1AF, or gene knockout approach, significantly inhibits the rate of migration in several cell types^37–41^. p38 is also involved in the migration of various cell types, such as smooth muscle cells, mammary epithelial cells, and neuronal cells^42–45^.

In this study, we aim to investigate how the modulation of STEAP2 levels affect copper concentrations in HCC cells and subsequently on MAPK signal transduction pathways as it pertains to cell migration and invasion, and whether reduction of STEAP2 protein expression can reverse the tumorigenic effect of STEAP2 in *vitro* and in *vivo*.

## Materials and methods

### Human tissue collection

Human HCC tumor and paired non-tumor liver tissues were collected from local Latino and non-Latino patients by the Transplant Center of the University of Texas Health Science Center at San Antonio for RNA extraction and RNA sequencing. Written informed consent was obtained from all participants. The study was approved by UT Health San Antonio Institutional Review Board and was performed in compliance with the Declaration of Helsinki and Good Clinical Practice guidelines.

### Cell lines and tumor specimens

Human HCC cell line SNU-398 and embryonic kidney 293T cell line were originally purchased from the American Type Culture Collection (ATCC; Manassas, VA). Human HCC cell line Huh7 (RRID: CVCL 0336) was a gift from Dr. Robert Lanford at the Texas Biomedical Research Institute in San Antonio, Texas. The HCC cell lines were authenticated with short tandem repeat (STR) assays and maintained in RPMI1640 medium (Cellgro 10-040-CV) supplemented with heat-inactivated 10% fetal bovine serum (FBS, Gemini Products Cat #900-108, Lot # A87F82H), 0.5% of 0.5 g/mL D-glucose stock, 1% of 1 mM sodium pyruvate, and 1% penicillin–streptomycin (10,000 ug/mL). Cells were maintained in 5% CO_2_ at 37 °C. Paired adjacent non-tumor and HCC tumor tissue were collected from Latino and non-Latino Caucasian patients by the Transplant Center at University Hospital (San Antonio, Tx) with written informed consent from the patients and approval by the Institutional Review Board (IRB), IRB number HSC20150834H. A small piece of tissue was formalin-fixed, paraffin-embedded for histology examination by a pathologist to confirm that the normal tissues did not contain tumor cells and the tumor tissues contained over 75% of tumor cells. The rest of the tissues were cut into small pieces, flash frozen in liquid nitrogen and stored in a liquid nitrogen tank. Tissue RNA extraction and sequencing procedures are provided in the supplementary file and reported previously^46^. Due to limited amount of tissue samples, quality of samples, and various assays performed, the number of samples used for the various assays are different as shown in the figures.

### Quantitative real-time RT-PCR

The mRNA level of *STEAP2* was measured by quantitative real-time reverse transcription-polymerase chain reaction (RT-qPCR). Total RNA (1 μg) was extracted from tissues or from HCC cells and was reverse-transcribed to cDNA using random primers and M-MLV reverse transcriptase from Invitrogen Life Technology (Grand Island, NY). Quantitative real-time PCR was performed using SYBR Green PCR Mix from Invitrogen Life Technologies. *STEAP2* primers (Forward: 5’CCAGTACCCAGAATCCAATGC3’; Reverse: 5’GAAATTCAACTGGCGGGCAAG3’) and GAPDH primers (Forward: 5’GCAGCCTCCCGCTTCGCTC3’; Reverse: 5’GCGCCCAATACGACCAAATCCGTT3’) used in this study were synthesized by Integrated DNA Technologies (Coralville, IA). GAPDH was used as an internal control.

### Reagents

Copper (II) sulfate pentahydrate was purchased from Sigma (Cat. # C8027). Cooper (II) Sulfate was dissolved in ddH2O and filtered with a 22 µm filter prior to use; a new solution was made for every single experiment. JNK inhibitor, SP600125 (Sigma, Cat. # S5567), and p38 alpha and p38 beta inhibitor, SB203580 (Sigma, Cat. # 559,395), were dissolved in DMSO. The human phospho-MAPK array kit was purchased from R&D Systems (Cat. # ARY002B, Minneapolis, MN).

### Protein array

Control and Knockdown cells were plated in 100 mm dishes, collected, and protein was extracted. The protein concentration was determined as described in western immunoblotting section. Eight hundred micrograms of total cell lysates from STEAP2 knockdown and matched control cells were incubated with membranes of the Human Phospho-MAPK Array Kit per the manufacturer’s instructions. Protein sample was incubated with each array at 4 °C overnight on a rocking platform shaker. The unbound proteins were removed, and the arrays were washed three times with washing buffer. Arrays were incubated with the primary antibody solution for 2 h at room temperature and then washed three times with a washing buffer. The secondary antibody solution was then added to the arrays on a rocking platform shaker for 1 h. The arrays were washed three times with washing buffer, and protein spots were visualized using the chemiluminescence detection reagents supplied in the Array Kit. Protein Array Analyzer for ImageJ^47^ was used to measure the intensity score of each duplicate array spot. The averaged intensity was calculated by subtracting the averaged background signal.

### Animal studies

Animal experiments were conducted following appropriate guidelines. They were approved by the Institutional Animal Care and Use Committee and monitored by the Department of Laboratory Animal Resources at the University of Texas Health Science Center at San Antonio. Animal health and behaviors were monitored daily. All xenograft-bearing mice had a single tumor and were euthanized by cervical dislocation after being anesthetized with 2% isoflurane inhalation.

Six-week-old male nude mice were used for in *vivo* animal experiments. The animals were housed under specific pathogen free conditions. SNU398 knockdown, overexpression, and control cells (2 × 10^6^ cells/100 µl/mouse) suspended in 50% Matrigel (Corning Life Sciences) and cold PBS were injected subcutaneously into the right flank of the mice. After tumor inoculation for 1–2 weeks, growing tumors were observed, and their size was recorded twice a week. The length and width of each tumor were measured using a caliper, and the volumes were calculated with the following formula: volume (mm^3^) = length x width x width/2. After 3–4 weeks, xenograft tumors were isolated from mice. The tissues were frozen for other experiments.

### Statistical analyses

Two-tailed Student t test was used to compare the means of two groups. One-way analysis of variance (ANOVA) with Tukey–Kramer post hoc test was used for analyzing data when means from more than two groups were compared. Results are expressed as mean ± SEM. Two-way ANOVA with Sidak’s multiple comparison test was used for analyzing data from growth curves. *P* < 0.05 was considered statistically significant.

## Results

### STEAP2 expression and copper levels are elevated in HCC tumor tissue

**Figure 1:**
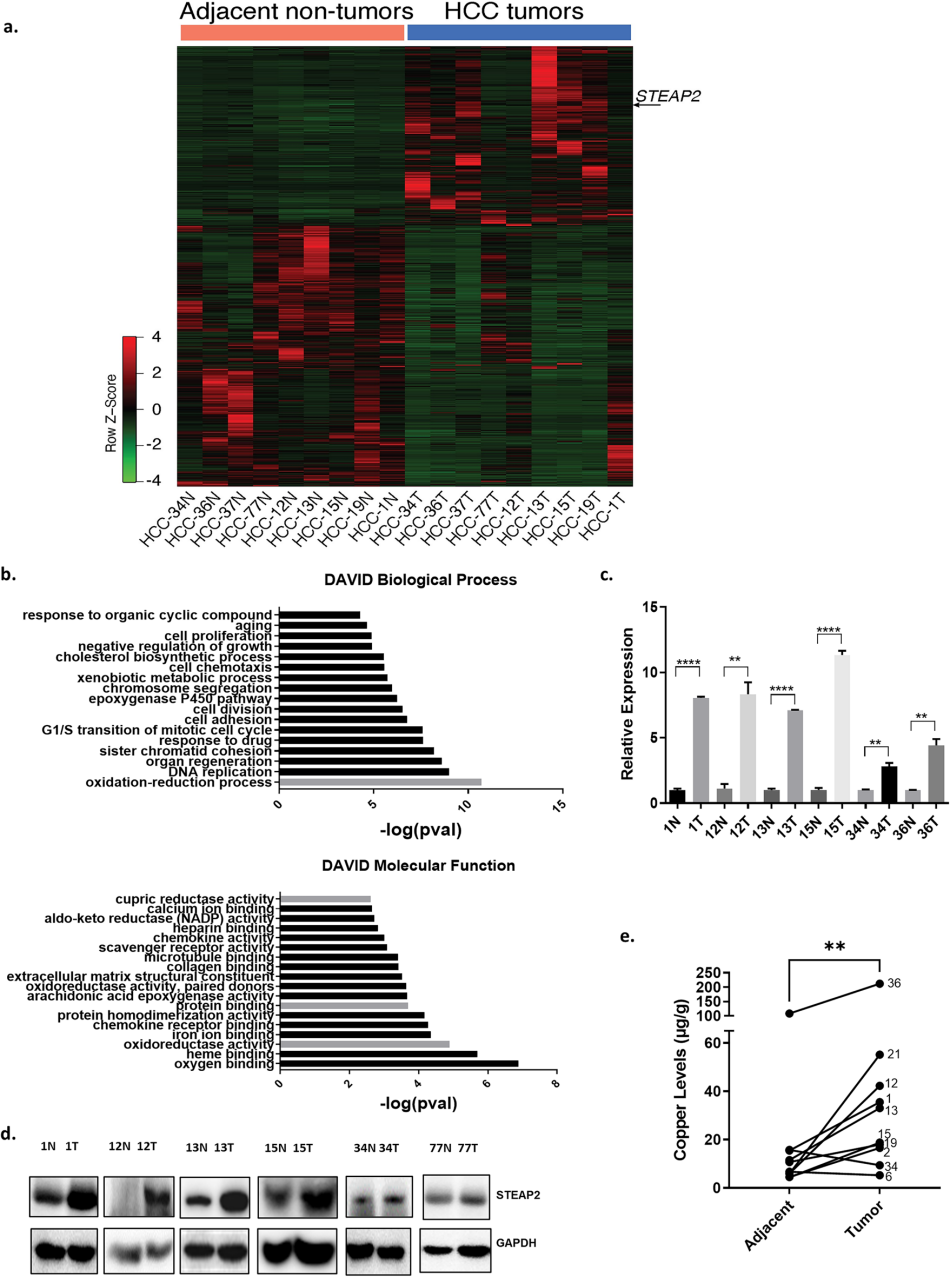
STEAP2 and Copper Levels are Elevated in HCC Tumor Tissue. (**a**) Heatmap showing unsupervised clustering of differentially expressed genes (1,288 genes with EdgeR Analysis) for 9 HCC tumor (T) and adjacent normal (N) samples from Hispanic/Latino patients. (**b**) DAVID Analysis with GO terms selected from top 20 biological process and molecular functions. Grey bars: GO terms that includes *STEAP2*. (**c**) *STEAP2* mRNA levels increased in HCC tumor (T) tissue compared to paired non-tumor (N) tissue from Hispanic/Latino patients measured by real time RT-PCR. Tumor *STEAP2* mRNA level was normalized by its paired adjacent non-tumor tissue STEAP2 mRNA level and is expressed as relative expression. (**d**) STEAP2 protein levels in paired HCC tumor (T) and adjacent non-tumor tissue (N) in Hispanic/Latino patients measured by Western blot. Western blot image was cropped to show STEAP2 protein band. E. Copper levels were measured in 10 paired samples via ICP-MS. *P < 0.05; **P < 0.01; ***P < 0.001. ****P < 0.0001 with unpaired T-test (**c**) or Wilcoxon test (**e**).

Demographics of nine Latino patients demonstrates that most patients had a tumor grade 2 and above, overweight, and have hepatitis C (Supplementary Table 1). Analysis with EdgeR and DESeq algorithms were used to obtain 1,288 differentially expressed genes for paired tumor and adjacent non-tumor tissues from the 9 Latino patients. Results are shown in the heatmap by unsupervised clustering of differentially expressed genes with STEAP2 indicated (Fig. 1a) (GSE202853). Functional assessment of these differentially expressed genes was performed using Database for Annotation, Visualization and Integrated Discovery (DAVID) to obtain the top biological processes and top molecular functions (Fig. 1b); a family of genes called *six transmembrane epithelial antigen of the prostate*, particularly *STEAP1* and *STEAP2*, were among the most common genes represented in the top categories (bars highlighted in grey—Fig. 1b). The top biological process and molecular function which include the STEAP family are oxidation–reduction process and oxidoreductase activity respectively. The gene *STEAP2* was found to have an average of eight-fold increase in tumor tissue compared to non-tumor tissue in the 9 cases (sup. Fig. 1a). Meanwhile, the gene *STEAP1* was found to have an average of five-fold increase in tumor tissue compared to non-tumor tissue in the 9 cases (not shown). STEAP2 was further investigated as a potential tumor target in HCC because STEAP1 does not have reductase activity by itself^48^. *STEAP2* overexpression in tumor tissue was confirmed via RT-qPCR in which all samples were significantly increased in tumor tissue compared to non-tumor tissue (Fig. 1c). Similar findings were seen in local non-Latino Caucasian patients (Sup. Fig. 1b). We also analyzed *STEAP2* gene expression in The Cancer Genome Atlas HCC (TCGA-LIHC) data, a dataset containing mostly non-Latino patients^49^, and found *STEAP2* expression was significantly higher in tumor tissue (*n* = 371) than in non-tumor tissue (*n* = 50) (Sup. Fig. 1c). More importantly, the expression of *STEAP2* was significantly associated with histologic grades (Sup. Fig. 1d). Patients expressing lower levels of *STEAP2* (in the lowest quartile; *n* = 92) exhibited a trend of improved overall survival compared to others (*n* = 278), although the difference was not statistically significant (logrank test *P* = 0.15) (Sup. Fig. 2a). A similar trend was observed when examining the combined expression of *STEAP2* and *STEAP1* (*P* = 0.097) (Sup. Fig. 2b). These findings demonstrate a potential link between STEAP2 and aggressiveness of liver HCC across multiple ethnic groups in the TCGA-LIHC dataset; a finding that appears to be more prominent when combining STEAP1 and STEAP2. The increased transcript levels of STEAP2 were found to translate to increased protein levels as demonstrated via Western blot (Fig. 1d) and immunohistochemistry (Sup. Fig. 1e).

Previous in vitro studies demonstrated that STEAPs function as a metalloredutase of iron and copper^11,14,19^ and facilitate the entry of these reduced ions into the cell. Iron overload leads to hereditary hemochromatosis and copper overload leads to Wilson disease, both are known risk factors for HCC, therefore, we sought to measure iron and copper levels in our paired tissue. Data from ten paired samples showed that iron levels in tumor and non-tumor samples were not significantly different (data not shown), however copper levels were markedly elevated in tumor tissue compared to non-tumor tissue (Fig. 1e). This is in accordance with previous studies on HCC and copper^50^. This finding suggests a potential link between STEAP2 and copper levels in HCC.

### STEAP2 knockdown decreases copper levels and inhibits growth in vitro and in vivo

**Figure 2:**
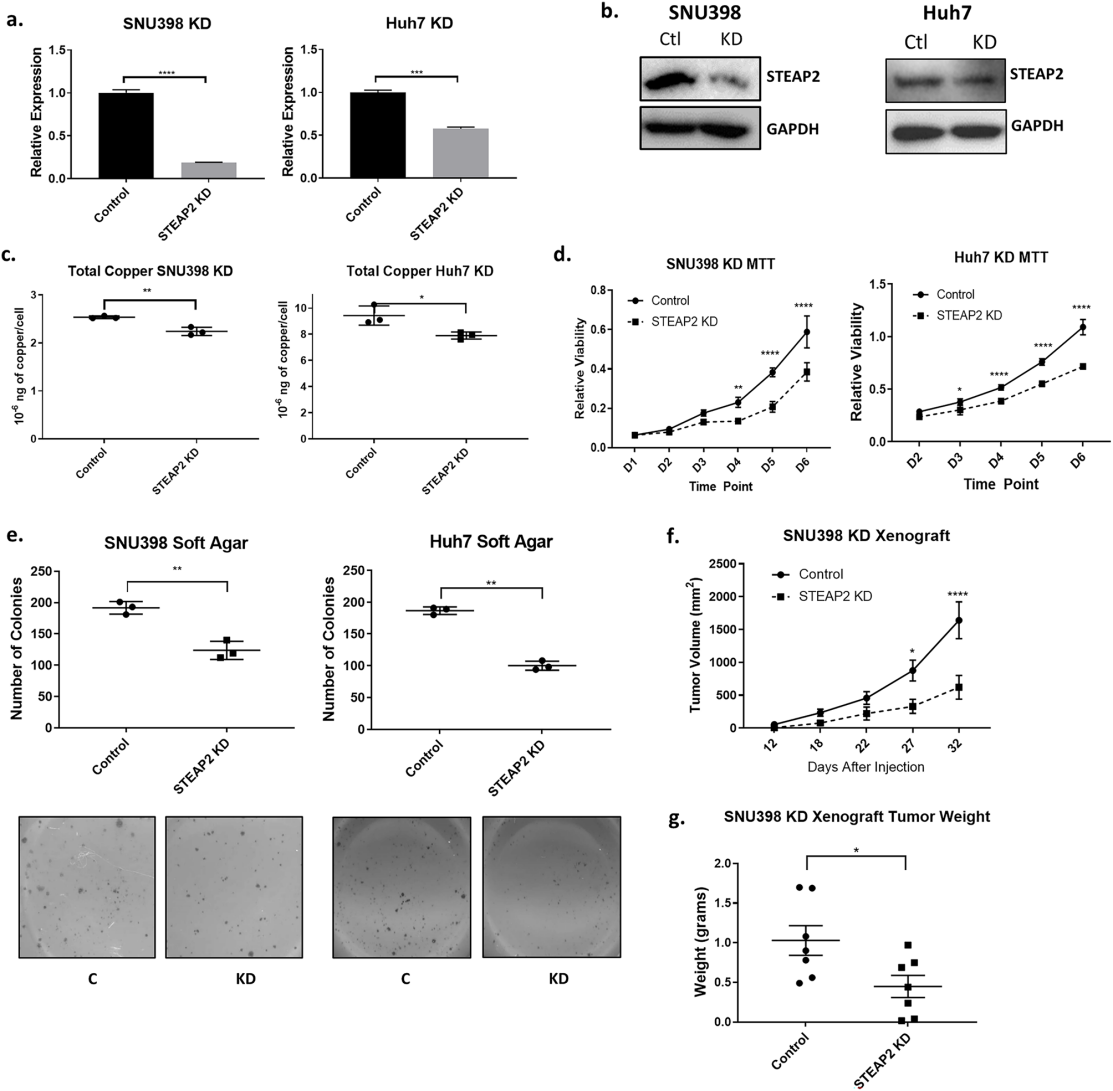
STEAP2 Knockdown Decreases Copper Levels and Inhibits Growth in vitro and in vivo. STEAP2 shRNA and matched control plasmid, TRC2-pLKO-puro vector, were transfected into SNU398 and Huh7 cells via lentiviral infection. (**a**) Confirmation of *STEAP2* stable knockdown in HCC cell lines in its transcription level with qRT-PCR. *GAPDH* transcript was used for normalization. (**b**) STEAP2 KD was confirmed at the protein level with Western blotting. Cells were harvested for protein and protein levels were measure with the STEAP2 antibody. GAPDH protein level was used to validate equal sample loading. Western blot image was cropped to show STEAP2 protein band. (**c**) Copper levels decreased in knockdown cells; measured via ICP-MS. STEAP2 knockdown inhibited HCC cell growth in an MTT assay (**d**) and anchorage independent growth in a soft agar assay (**e**). (**f**) Growth curve of tumors formed by Control or STEAP2 shRNA-transfected SNU398 cells in male nude mice. Tumor volume was calculated using the formula: v = length x width x width × 0.5. Each data point represents the mean ± SEM of seven tumors. (**g**) Tumors excised from euthanized mice were weighed at the end of the experiment. Each data point represents a tumor with Mean ± SEM also presented. *P < 0.05; **P < 0.01; ***P < 0.001. ****P < 0.0001 with unpaired T-test or two-way ANOVA.

To determine the function of STEAP2 in HCC, its expression was knocked down in two HCC cell lines, SNU398 and Huh7. STEAP2 gene expression was knocked down in stable cell lines by 80% and 50% respectively in the two cell lines (Fig. 2a), which translated to a similar respective reduction in its protein levels (Fig. 2b). Transient STEAP2 knockdown (Sup. Fig. 3 a,b) with siRNA#1 (targeting open reading frame), siRNA#2 (targeting 3’ untranslated region), or a pooled siRNA (sipool) demonstrated gene expression levels decreased by 30–60%; slightly less effective than the stable cell lines. Copper levels were found to be significantly decreased in STEAP2 knockdown cells (Fig. 2c); this substantiates our finding that tumor tissue with elevated STEAP2 levels has higher copper levels compared to non-tumor tissue with lower STEAP2 levels. Stable STEAP2 knockdown cells exhibited decreased growth in MTT viability assay (Fig. 2d) and cell confluency assay (Sup. Fig. 3f). Similarly, transient knockdown with siRNA#2 or sipool also decreased growth in cell confluency assay and MTT assay (Sup. Fig. 3c) respectively. Furthermore, STEAP2 knockdown cells grew less colonies in a soft agar anchorage independent growth assay (Fig. 2e). More significantly, knockdown of STEAP2 significantly reduced the growth of tumors formed by subcutaneously injected SNU398 cells in nude mice (Fig. 2f). The mean weight of the tumors from mice injected with *STEAP2* knockdown cells were lower than that of the tumors from mice injected with control cells (Fig. 2g). To determine the cause of STEAP2 knockdown’s effect on decreased cell and tumor growth, cell cycle analysis in Huh7 and SNU398 control and KD cells revealed no significant difference in SNU398 cells in response to knockdown (data not shown). This suggests that STEAP2’s effect on reduced cell growth is not attributed to an altered cell cycle profile. Furthermore, no significant increase of apoptosis was found in *STEAP2* knockdown cells (data not shown). Further studies are required to examine the exact mechanism by which STEAP2 is altering proliferation.

### Ectopic overexpression of STEAP2 increases copper levels and promotes growth in vitro and in vivo

To further explore the role of STEAP2 in HCC cells, we overexpressed STEAP2 in Huh7 and SNU398 cells, which was confirmed by RT-qPCR and Western blot (Sup. Fig. 4a,b). STEAP2 overexpression cells had significantly higher copper levels compared to control cells (Sup. Fig. 4c). As expected, STEAP2 overexpression cells showed increased growth on plastic (Sup. Fig. 4d) and in soft agar (Sup. Fig. 4e). Overexpression of *STEAP2* increased the growth of tumors formed by subcutaneously injected SNU398 cells in nude mice (Sup. Fig. 4f,g). These findings corroborate with the results of *STEAP2* knockdown, thus demonstrating a tumor-promoting activity of STEAP2 in HCC cells.

### Manipulation of STEAP2 expression reveals its role in chemotaxis, cell adhesion, migration, and invasion

**Figure 3:**
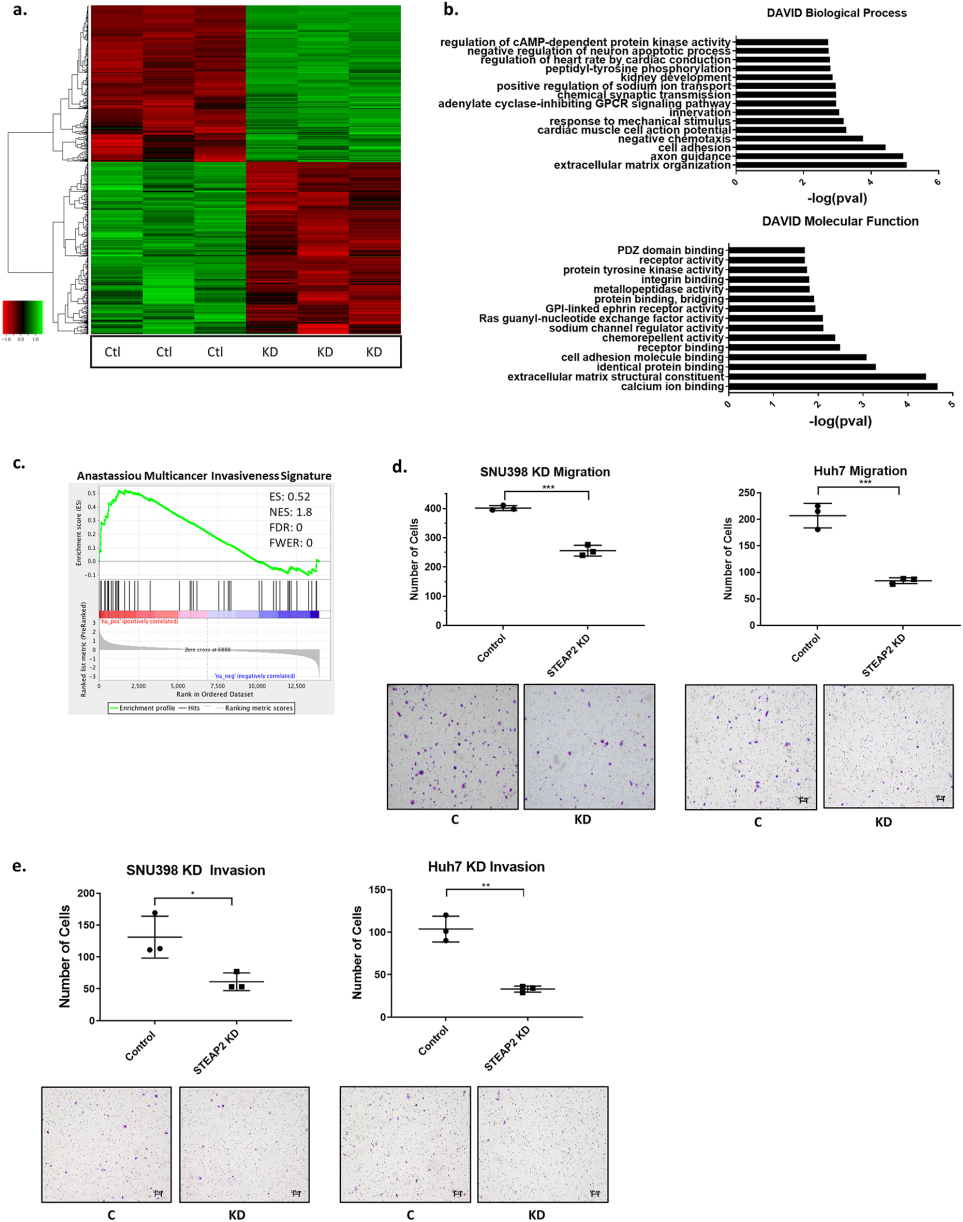
Manipulation of STEAP2 Expression Reveales its Role in Chemotaxis, Cell Adhesion, Migration, and Invasion. (**a**) Heatmap showing significantly differentially expressed genes upon *STEAP2* knockdown. Three samples of STEAP2 knockdown were compared to three matched control samples. Color bar on the left side indicates genes that are upregulated (green) and downregulated (red) upon *STEAP2* knockdown. Expression levels were scaled so that green indicates relatively higher expression whereas red indicates lower expression. (**b**) Gene Ontology analysis of differentially expressed genes with the Database for Annotation, Visualization and Integrated Discovery (DAVID) identified top Biological Processes (top) and top Molecular Functions (bottom) that are enriched upon *STEAP2* knockdown. (**c**) Gene set enrichment analysis. Genes were rank ordered according to their fold change between control and *STEAP2* knockdown with genes highly expressed in control on the left side. A set of invasiveness signature genes reported by Anastassiou and co-workers^51^ was analyzed and indicated as black bars in the plots. The invasiveness signature genes were significantly enriched in control cells and decreased in STEAP2 knockdown cells. ES, enrichment score. NES, normalized enrichment score. FDR, false discovery rate, FEWR, familywise error rate *P*-value. *STEAP2* Knockdown inhibited HCC cell migration (**d**) and invasion (**e**) in transwell assay. *P < 0.05; **P < 0.01; ***P < 0.001 with unpaired T-test.

To investigate potential mechanisms of the tumor-promoting activity of STEAP2, we performed whole genome RNA sequencing on the SNU398 control versus knockdown cells (triplicates) and used the Deseq algorithm to estimate the differential expression in read counts and their statistical significance. We obtained 514 differentially expressed genes as is shown in the heatmap (Fig. 3a). Further analysis of these differentially expressed genes with DAVID revealed that the most significant Biological Processes and Molecular Functions associated with the differentially expressed genes were related to extracellular matrix organization, cell adhesion, and negative chemotaxis (Fig. 3b). Interestingly, an invasiveness signature gene set^51^ was significantly enriched in the control cells relative to *STEAP2* knockdown cells (Fig. 3c). The list of genes included in the invasiveness signature genes are listed in Supplementary Table 2; the genes enriched in the control cells (Log_2_FC ≥ 0.5) are highlighted in green. In accordance with these results, *STEAP2* stable knockdown decreased the number of cells that migrated in Transwell by 67% in SNU398 and by 62% in Huh7 cells (Fig. 3d). Similar decrease of migration was also observed in transient knockdown HCC cells with either *STEAP2* siRNA #2 or *STEAP2* siRNA pool (Sup. Fig. 3d,e). Invasion assay also showed that stable *STEAP2* knockdown reduced the number of cells that invaded through Matrigel-coated Transwell by 60% in SNU398 and Huh7 cells (Fig. 3e). Both SNU398 and Huh7 overexpression cells showed increased migration by about 35% and invasion by 30–40% (Sup. Fig. 4h,i). These results demonstrate that STEAP2 expression increases cell migration and invasion in both SNU398 and Huh7.

### STEAP2 knockdown decreases JNK and p38 phosphorylation

**Figure 4:**
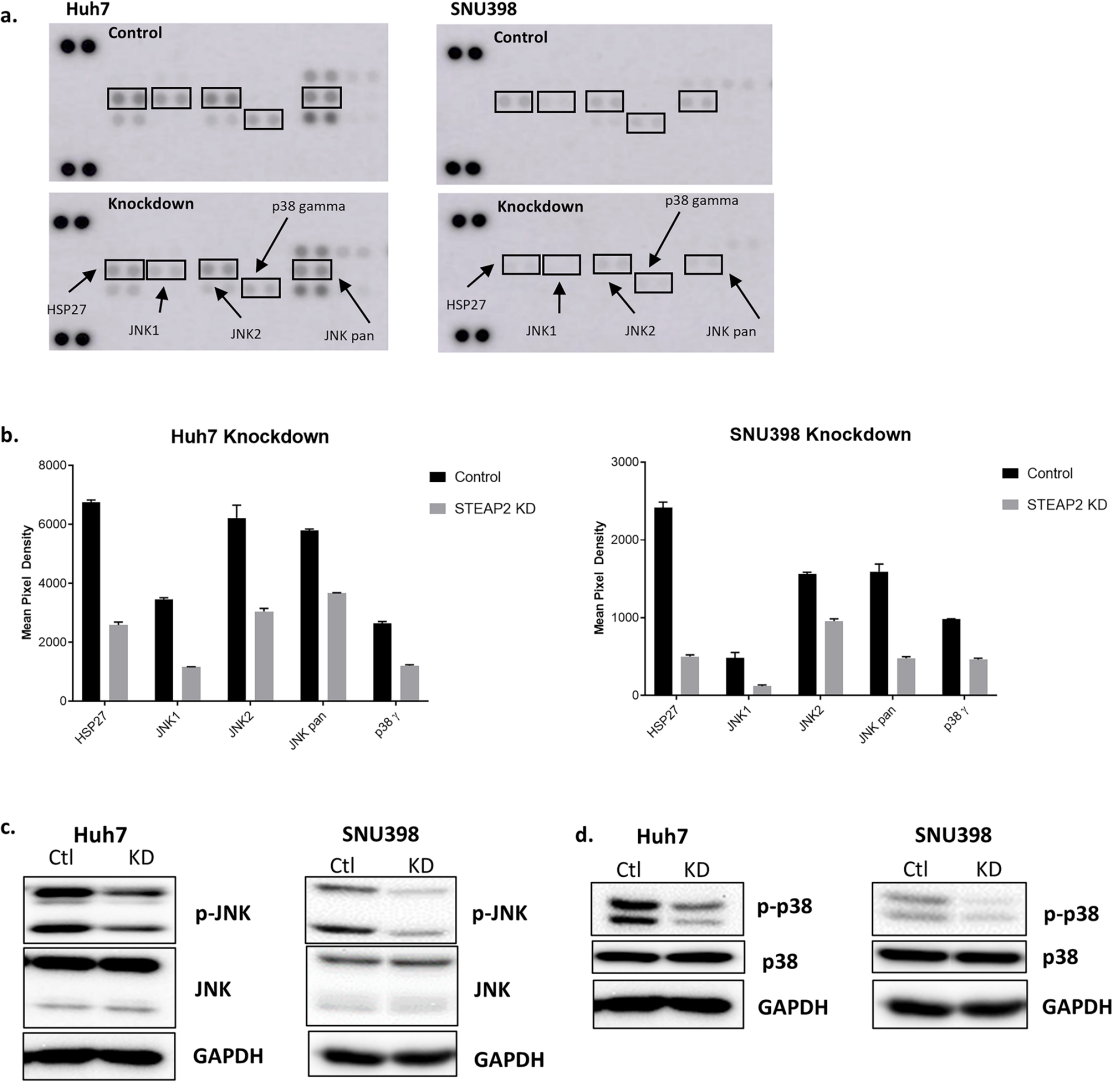
STEAP2 Knockdown Decreases JNK and p38 phosphorylation. (**a**) The results of the phospho-MAPK protein array analysis using *STEAP2* knockdown and control cells. Cell lysates were assessed using the Proteome Profiler Human Phospho-MAPK Array membranes (ARY003B, R&D Systems) overnight at 4 °C. Biotinylated detection antibodies were applied, and membranes were visualized using chemiluminescence. (**b**) The graph shows the pixel intensity of proteins with significant difference in STEAP2 knockdown (KD) cells compared to matched control cells. Densitometry analysis was performed using the Protein Array Analyzer for ImageJ. The decreased phosphorylation patterns of JNK isoforms at T183/Y185 (**c**) and p38 isoforms at T180/Y182 (**d**) due to STEAP2 KD were validated by Western blot. Western blot image was cropped to show appropriate protein band (SNU398 blot cropped). GAPDH protein level was used to validate equal sample loading.

In prostate cancer cells, STEAP2 expression was required for optimal ERK activity; phosphorylation of ERK was strongly downregulated in STEAP2 knockdown cells^15^. We explored this potential pathway as the mechanism by which STEAP2 promotes or aids in the progression of HCC. There was no significant difference in the phosphorylated ERK in control and knockdown cells (data not shown). Therefore, we performed a phospho-MAPK protein array analysis on STEAP2 control and knockdown cells. We found that phospho-JNK and phospho-p38 were significantly decreased in STEAP2 knockdown cells (Fig. 4a,b). These findings were confirmed via Western blot in both cell lines (Fig. 4c,d).

### Copper supplementation activates JNK and p38, and rescues migration of STEAP2 knockdown cells

STEAP2 is known to have cupric reductase activity; HCC cell lines with STEAP2 knockdown showed a decrease in copper levels (Fig. 2c) whereas STEAP2 overexpression cells showed an increase in copper levels (Sup. Fig. 3c). Next, we examined if copper supplementation affects STEAP2 protein levels and the phosphorylation of JNK and p38. Treatment of SNU398 and Huh7 cells with 19.5 µM of copper (II) sulfate pentahydrate for 24 h did not change STEAP2 levels (Sup. Fig. 5a). Since RPMI 1640 medium used for culturing HCC cell lines contains no source of copper^52^ but fetal bovine serum contains copper at a concentration of 2.5 µM^53^, we opted to serum-starve the cells for 48 h prior to the copper treatment for 24 h. There was no significant change in STEAP2 levels with serum-starvation (Sup Fig. 5b). On the other hand, treatment with copper (II) sulfate for 24 h increased phosphorylated JNK and p38 (Sup. Fig. 5c) in a dose dependent manner in SNU398 cells and Huh7 (Sup. Fig. 5d).

**Figure 5:**
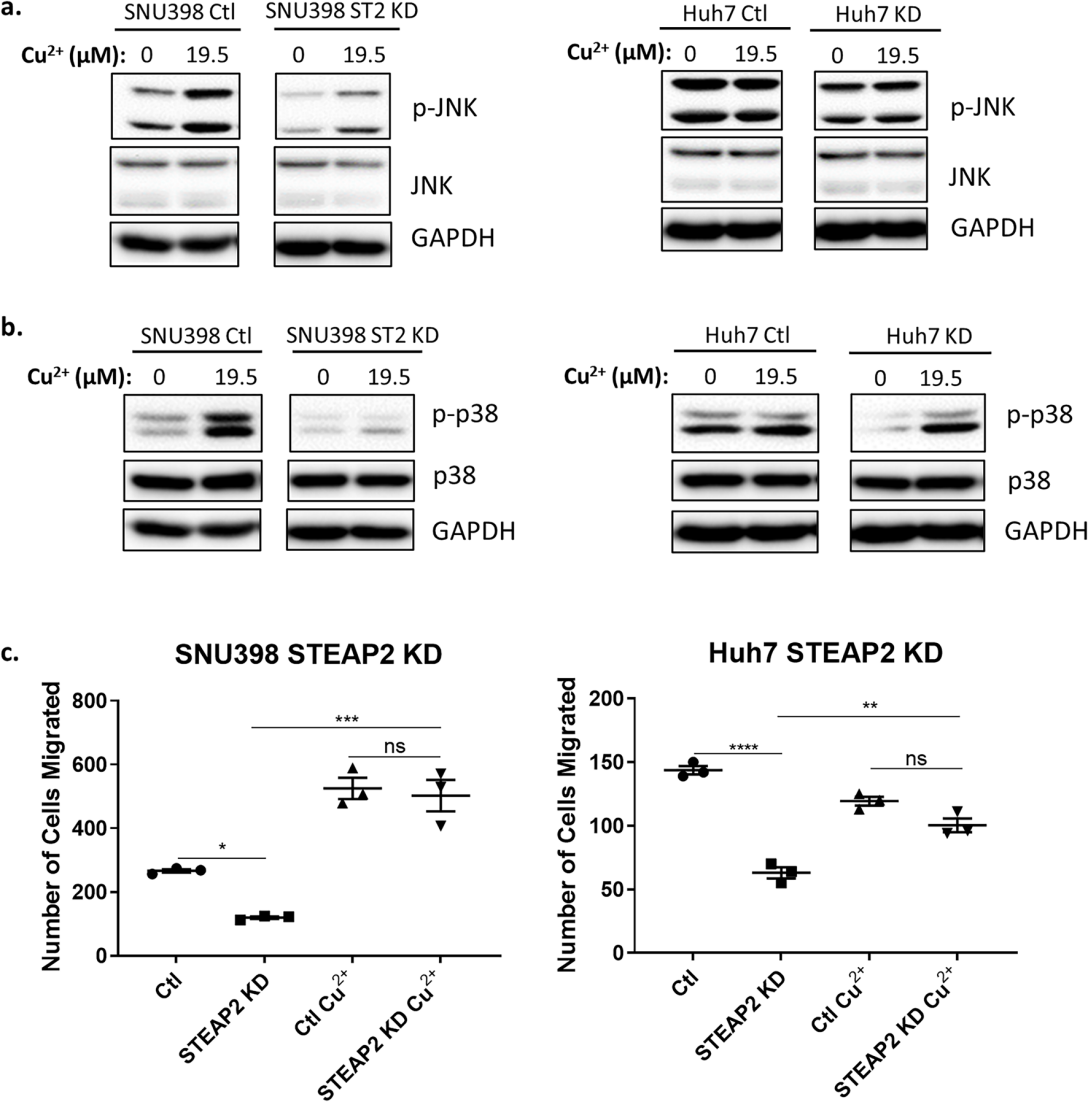
Copper Supplementation Rescues STEAP2 Knockdown Effect on Migration via JNK and p38 Phosphorylation. Copper (II) sulfate pentahydrate supplementation (19.5 µM) increases phosphorylation of JNK isoforms at T183/Y185 (**a**) and p38 isoforms at T180/Y185 (**b**) in *STEAP2* knockdown cells detected by Western blot. Western blot image was cropped to show appropriate protein band. (**c**) Copper (II) supplementation (19.5 µM) rescues the number of cells migrated in *STEAP2* KD cells. *P < 0.05; **P < 0.01; ***P < 0.001. ****P < 0.0001 with one-way ANOVA.

We then tested copper supplementation in the STEAP2 knockdown cells and found that copper supplementation increased phosphorylation of JNK and p38 in both control and *STEAP2* knockdown SNU398 cells (Fig. 5a-b). Copper treatment for 24 h led to a significant increase in migration in *STEAP2* knockdown cells (Fig. 5c), suggesting that copper rescued the knockdown effect on migration. Interestingly, copper supplementation did not stimulate the migration of Huh7 control cells but significantly stimulated the migration of SNU398 control cells (Fig. 5c). The cause for different responses in these two cells lines is not known, however, one possible explanation is that Huh7 control cells have higher levels of copper compared to SNU398 control cells (Fig. 2c), suggesting that a threshold level of copper might have been reached in Huh7 cells. This assumption is consistent with the observation that treatment with copper increased the levels of phosphorylated JNK and p38 in the SNU398 control cells but not in the Huh-7 control cells (Fig. 5a,b). These findings suggest that reduced copper levels in *STEAP2* knockdown cells led to decreased phosphorylation of JNK and p38, subsequently resulting in a decrease in migration.

### Inhibition of JNK and/or p38 decreases migration in HCC cell lines

**Figure 6:**
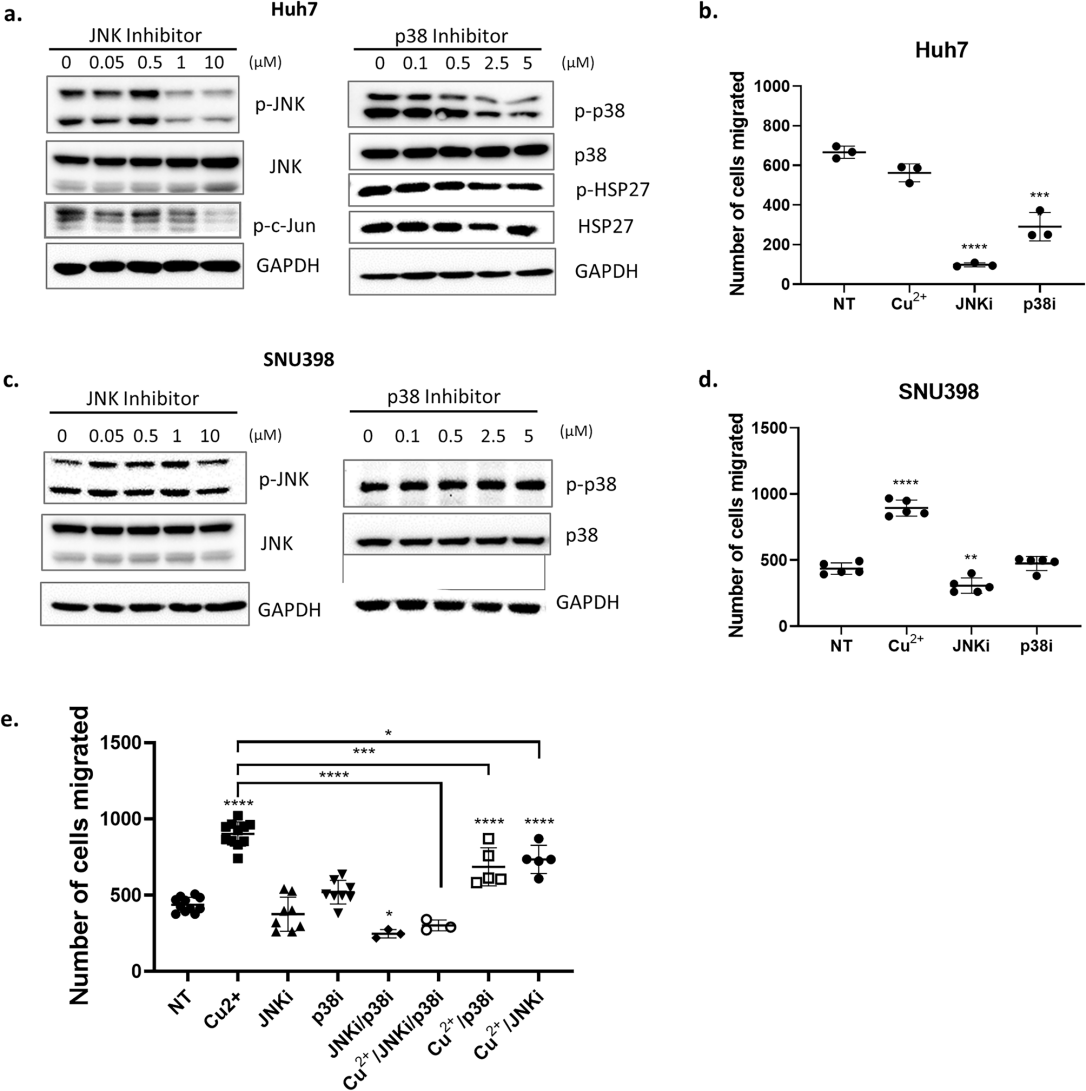
JNK Inhibitor and p38 Inhibitor Decrease Migration in HCC cell lines. Huh7 cells (**a**) and SNU398 cells (**b**) were plated in 60 mm dishes at a density of 8 × 10^5. They were treated next day for 24 h with different concentrations of the inhibitor as shown in the figure before cells were collected for Western blot. For the transwell migration assay, p38 inhibitor (5 µM) or JNK inhibitor (10 µM) was added in both top and bottom wells. (**c**) SNU398 cells were treated with copper (19.5 µM), p38 inhibitor (5 µM), JNK inhibitor (10 µM), and/or the combination for 24 h, followed by migration assay in transwells. NT: no treatment. Western blot image was cropped to show appropriate protein band. Each bar is mean ± SD. *P < 0.05; **P < 0.01; ***P < 0.001; ****P < 0.0001 with one-way ANOVA.

To further delineate the specific roles of copper, JNK and p38 in migration, we treated Huh7 cells with JNK inhibitor, SP600125, and p38 alpha and p38 beta inhibitor, SB203580. Treatment of Huh7 cells with JNK or p38 inhibitor significantly decreased phosphorylated JNK or p38 respectively (Fig. 6a), in addition to cell migration (Fig. 6b). In contrast, JNK and p38 inhibitors showed limited effect on the phosphorylation (Fig. 6c) and migration (Fig. 6d) of SNU398 cells. This is likely due to higher levels of phosphorylated JNK and p38 in Huh-7 cells than in SNU398 cells (Fig. 5a,b). Since copper supplementation stimulated phosphorylation of JNK and p38 in SNU398 cells (Fig. 5a,b) and significantly increased their migration (Fig. 5c,d), we examined whether the copper-mediated cell migration was due to JNK and/or p38 activation. Treatment with JNK or p38 inhibitor significantly reduced copper-stimulated cell migration, which was completely abolished when the cells were treated with both JNK and p38 inhibitors (Fig. 6c). Thus, STEAP2 promotes HCC cell migration and invasion at least in part by stimulating copper-mediated activation of JNK and p38.

## Discussion

In this study, we demonstrate that STEAP2 plays a significant role in HCC cell growth, migration, and tumor progression by performing various rigorous in vitro and in vivo assays. More significantly, we identified a novel mechanism that mediates the tumor-promoting activity of STEAP2 in HCC.

STEAP2 expression is significantly increased in HCC of Latinos and NLW patients in South Texas and in NLW patients from the TCGA database, as shown in our study and the latter shown by Fu et al.^22^, suggesting a tumor-promoting role in HCC. Due to the small number of patient samples in this study, no definite correlation between STEAP2 and increasing tumor grade was established, as has been demonstrated in prostate cancer studies in which STEAP2 expression significantly correlated with Gleason score^20^. However, analysis of tumor samples from the TGCA database revealed a strong correlation between *STEAP2* expression and increasing tumor grade and a trend of improved survival in patients expressing lower levels of *STEAP2.* While STEAP2 alone is not a strong prognostic marker of HCC, its significant role in driving HCC cell proliferation, migration, and invasion suggests that it collaborates with other molecules and pathways to promote HCC malignancy. Thus, it is possible that its prognostic power may improve when combined with other HCC-promoting molecules in a clinical setting, which requires further investigation. Furthermore, our study showed noticeably higher upregulation of STEAP2 expression in Hispanic/Latino patients compared to South Texas NLW patients with HCC, and NLW patients with HCC in the TCGA database. Further studies are needed to determine whether this difference contributes to the high incidence of HCC in the Latino population.

By reducing the protein expression of STEAP2 in HCC cell lines, we demonstrate that STEAP2 plays a role in cell growth and migration/invasion in vitro and in vivo. Studies on prostate cancer have shown similar findings^15,20^, while studies on breast cancer have demonstrated opposite findings^21^; this highlights the need for more comprehensive analyses of the role of STEAP2 in cancer. In addition, there are few mechanistic studies demonstrating the relationship between STEAP2 and malignant properties of other types of tumors. In prostate cancer, Burnell et al. demonstrated that PC3^KD^ cells, but not LNCaP^KD^ cells, decreased proliferation with no corresponding significant differences in cell cycles phases^20^. This contrasts with Wang et al. who showed a significant decrease in proliferation of LNCaP^*KD*^ cells, attributed to partial cell cycle arrest in G0-G1 and a corresponding decrease in cells in the S phase^15^. In our study, no significant differences in cell cycle phases between HCC lentiviral control and knockdown cells were observed, suggesting that cell cycle profile may not be the only mechanism for the reduced proliferative capacity in STEAP2 knockdown cancer cells. This is in accordance with findings from Burnell et al.^20^. Another explanation for the cell growth reduction in STEAP2 knockdown cells might be due to increased apoptosis. Wang et al. showed that knockdown of STEAP2 strongly increased apoptosis in prostate cancer cells, however, the apoptotic pathway in which STEAP2 exerts its effect is not currently understood^15,54^. In our study, we did not find any significant increase in apoptosis in STEAP2 knockdown HCC cells. In the prostate cancer study, apoptosis was induced by tumor necrosis factor-related apoptosis inducing ligand (TRAIL)^15^, however, in our study the only form of induction we used was serum starvation for 24 h. Perhaps this was insufficient to induce apoptosis to observe a difference in control and STEAP2 knockdown cell lines.

The migratory and invasion potential of STEAP2 knockdown cells was shown to be significantly reduced when compared to control cells by about 60–70% for the two HCC cell lines. This level of reduction in invasion is higher than that seen in prostate cancer cells (20–50% reduction) by Burnell el al.^20^. Moreover, the RNA sequencing data from control and knockdown cells highlighted migration related biological processes and molecular functions. GSEA also demonstrated that invasiveness signature genes were significantly enriched in control cells and decreased in STEAP2 knockdown cells. STEAP2 overexpression increased migration and invasion by 30–40% which was slightly less than was reported in STEAP2 transfected PNT2 cell lines (normal prostate epithelium)^54^. These findings suggest that STEAP2 plays a significant role in promoting cancer cells to invade the local microenvironment, leading to tumor metastasis.

To validate the role of STEAP2 in tumor progression, we overexpressed STEAP2 in Huh7 and SNU398 cells by creating stable overexpression cell lines. STEAP2 overexpression cells demonstrated increased 2-dimensional and 3-dimensional growth in soft agar in vitro and increased xenograft growth in vivo. Furthermore, STEAP2 overexpression cells demonstrated increase migration and invasion; this is in accordance with findings from Whiteland et al. in which upregulated STEAP2 in PNT2 cells caused an increase in migration compared to non-transfected PNT2 cells^54^. The STEAP2 overexpression in HCC cells was lost as cells were passaged, which limited further experiments. Perhaps this was due to the strict copper homeostasis that is required in HCC cells. Further characterization of STEAP2 overexpression HCC cells will need to be carried out in future studies.

We demonstrate that STEAP2 knockdown cells have reduced copper levels, while STEAP2 overexpression cells have increased copper levels. Previous literature has shown that STEAP2 functions as a cupric reductase via in vitro studies^11^. In prostate cancer, STEAP2 expression was associated with ERK activity; phosphorylation of ERK was increased on ectopic expression of STEAP2 and was strongly downregulated in STEAP2 knockdown cells^15^. In HCC cells, phospho-ERK was not decreased in knockdown cells, but the phosphorylation of the stress-activated MAPKs, JNK and p38, were consistently decreased in our knockdown cells. Accumulating evidence suggests that the JNK and p38 pathways are involved in the regulation of cell migration^33–35^; this is in accordance with our findings that STEAP2 knockdown cells exhibit strong inhibition of migration and invasion. Moreover, JNK and p38 have been reported to be activated by copper^30^. This is consistent with our findings, copper supplementation increased phosphorylation of JNK and p38 in HCC cells. Thus, the decreased levels of copper in STEAP2 knockdown cells contributed to the decreased phosphorylation of JNK and p38, thus the inhibition of migration and invasion.

Copper can induce migration via other mechanism, such as increasing copper-dependent LOX; LOXL2 and LOXL3 interact with Snail which diminishes GSK3β-mediated phosphorylation of Snail, thereby stabilizing Snail, inhibiting E-cadherin expression, and favoring the mesenchymal phenotype^55,56^. RNA sequencing data from our STEAP2 knockdown study demonstrates that LOXL2 is also significantly decreased in knockdown cells. Therefore, copper activation of JNK and p38 may not be the only mechanisms that drive migration and invasion in our HCC cells.

In conclusion, our data suggest that STEAP2 contributes to HCC progression by increasing copper absorption in hepatocytes, activating stress-activated MAPK pathways JNK and p38, and inducing migration and invasion in HCC cells. This investigation highlights STEAP2 as a potential prognostic biomarker for advanced HCC.

### Supplementary Information

Supplementary Information 1.

Supplementary Information 2.

## Data Availability

All data generated or analyzed during this study are included in this published article and its supplementary information files except that the RNA sequencing data are deposited at GEO database, Accession #GSE202853.

## Abbreviations

FBS: Fetal bovine serum
STEAP2: Six transmembrane epithelial antigen of prostate 2
HCC: Hepatocellular carcinoma
KD: Knockdown
OE: Overexpression
TBST: Tris buffered saline with tween
DAVID: Visualization and integrated discovery
TCGA: The cancer genome atlas
TRAIL: Tumor necrosis factor-related apoptosis inducing ligand
