# Supplementary material for: STEAP2 promotes hepatocellular carcinoma progression via increased copper levels and stress-activated MAP kinase activity
Source: Sci Rep. 2024 Jun 3;14:12753. doi: 10.1038/s41598-024-63368-2 (PMC11148201; doi:10.1038/s41598-024-63368-2)
Supplement: Supplementary file 1 — Supplementary Information 1. [file 41598_2024_63368_MOESM1_ESM.pdf]

Original Western Blots

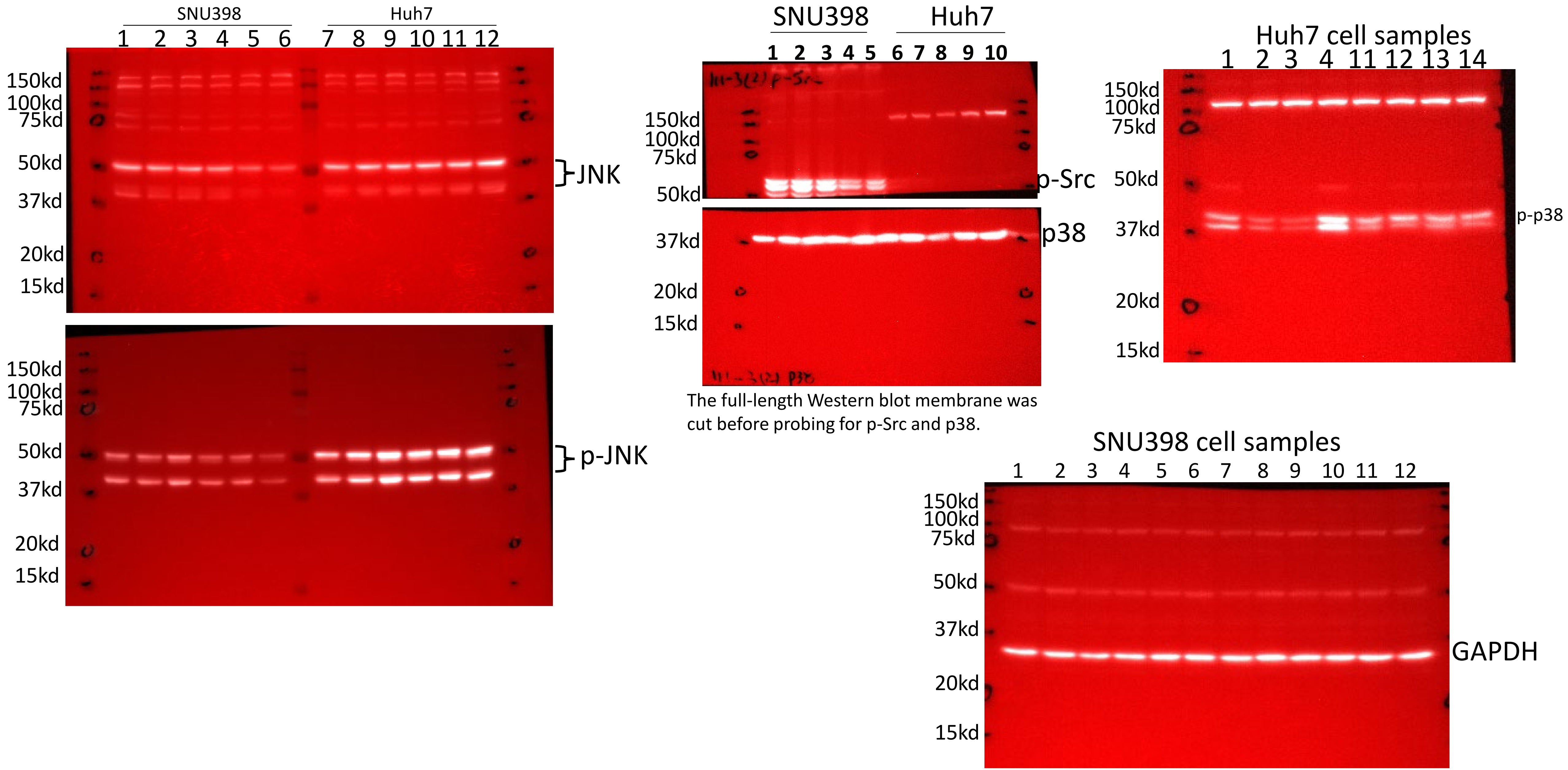

The full-length Western blot membrane was cut before probing for p-Src and p38.

Full-length Western blots illustrating the positions of JNK, p-JNK, p38, p-p38, and GAPDH bands using cell lysate from samples not related to this study. In this study, for the detection of these proteins, Western blot membranes were often cut above and below 37-50 KD molecular markers before they were blotted with various primary and secondary antibodies. The red background images are shown because the images of the molecular weight markers were better captured with the red background images than with the black and white images.

Fig 1D

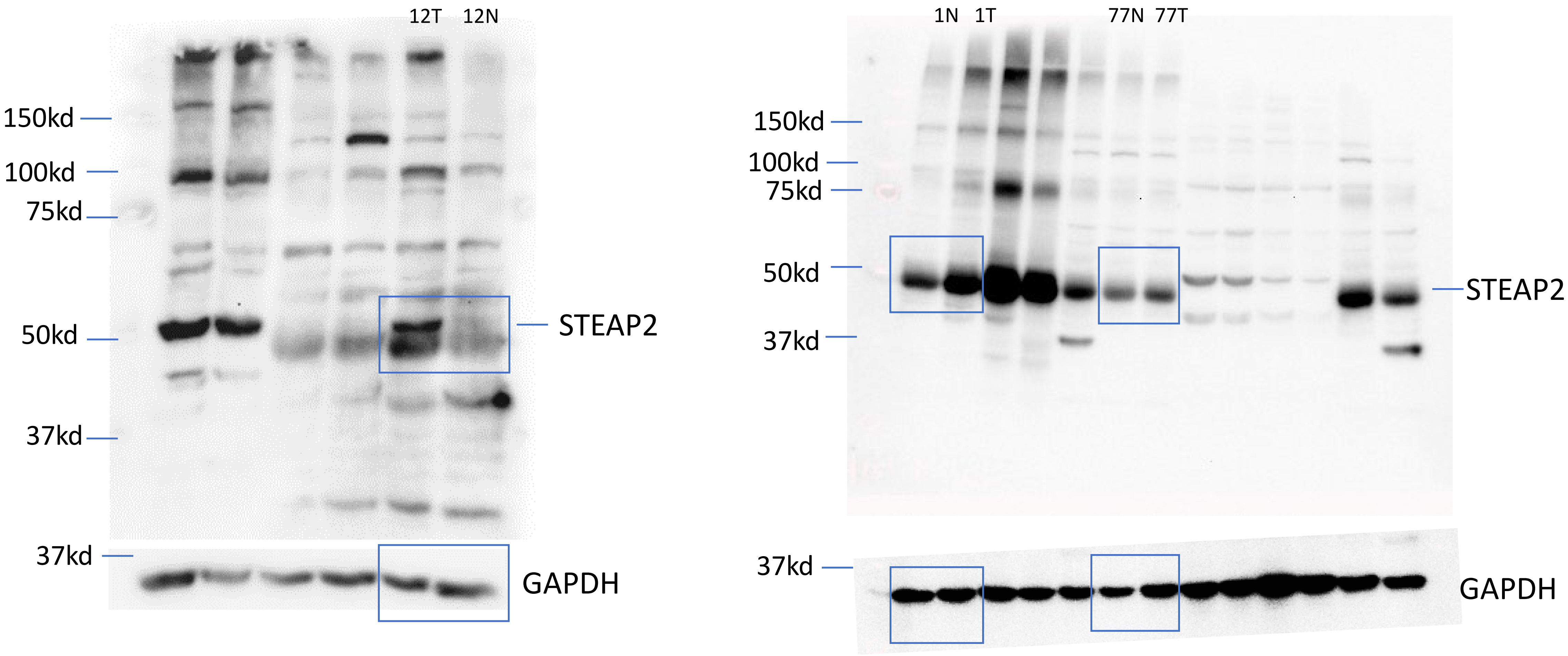

STEAP2 was first probed in the full membrane. The membrane below 50kd marker was then cut and probed for GAPDH.

Fig 1D

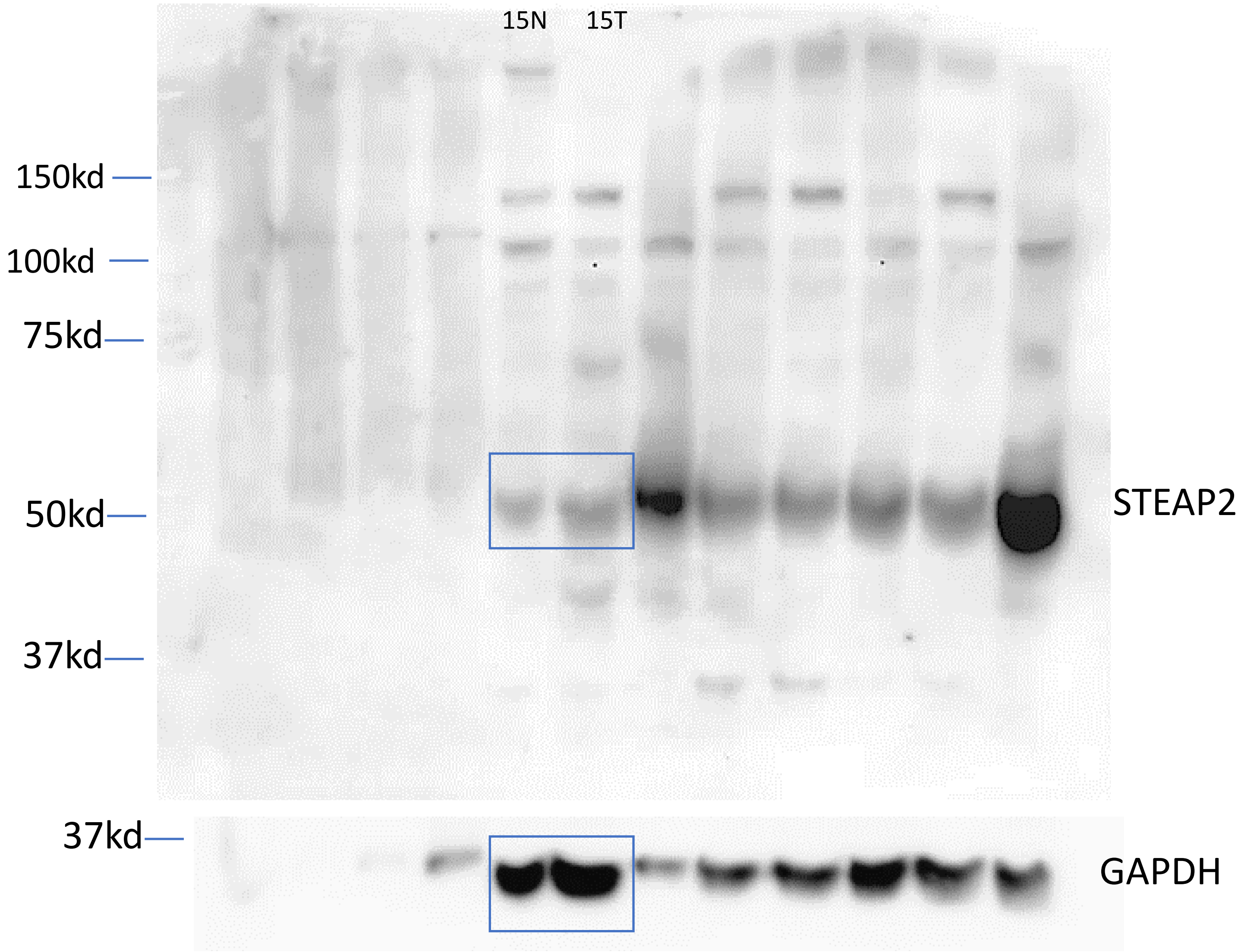

STEAP2 was first probed in the full membrane. The membrane below 50kd marker was then cut and probed for GAPDH.

Fig 1D

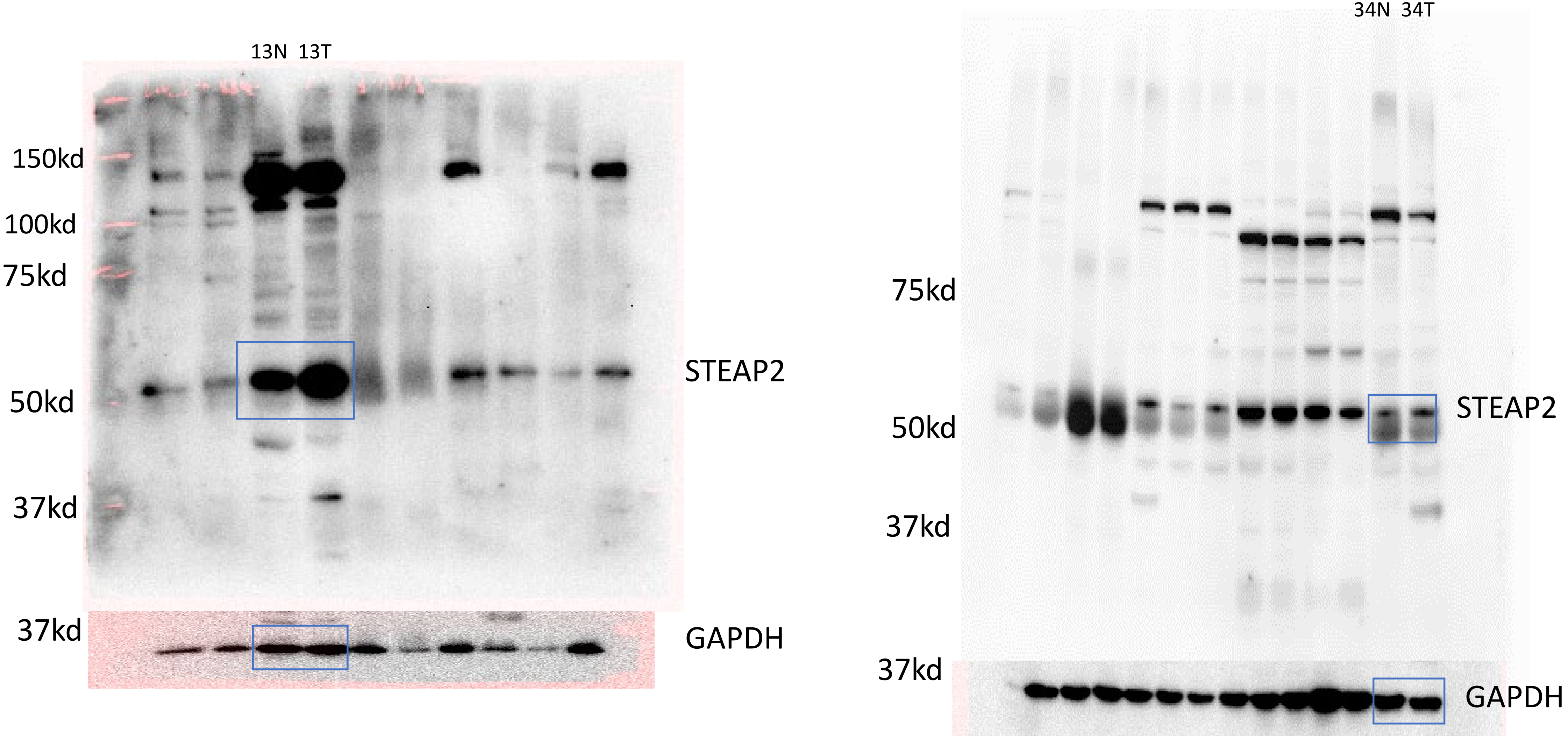

STEAP2 was first probed in the full membrane. The membrane below 50kd marker was then cut and probed for GAPDH.

Fig 2B

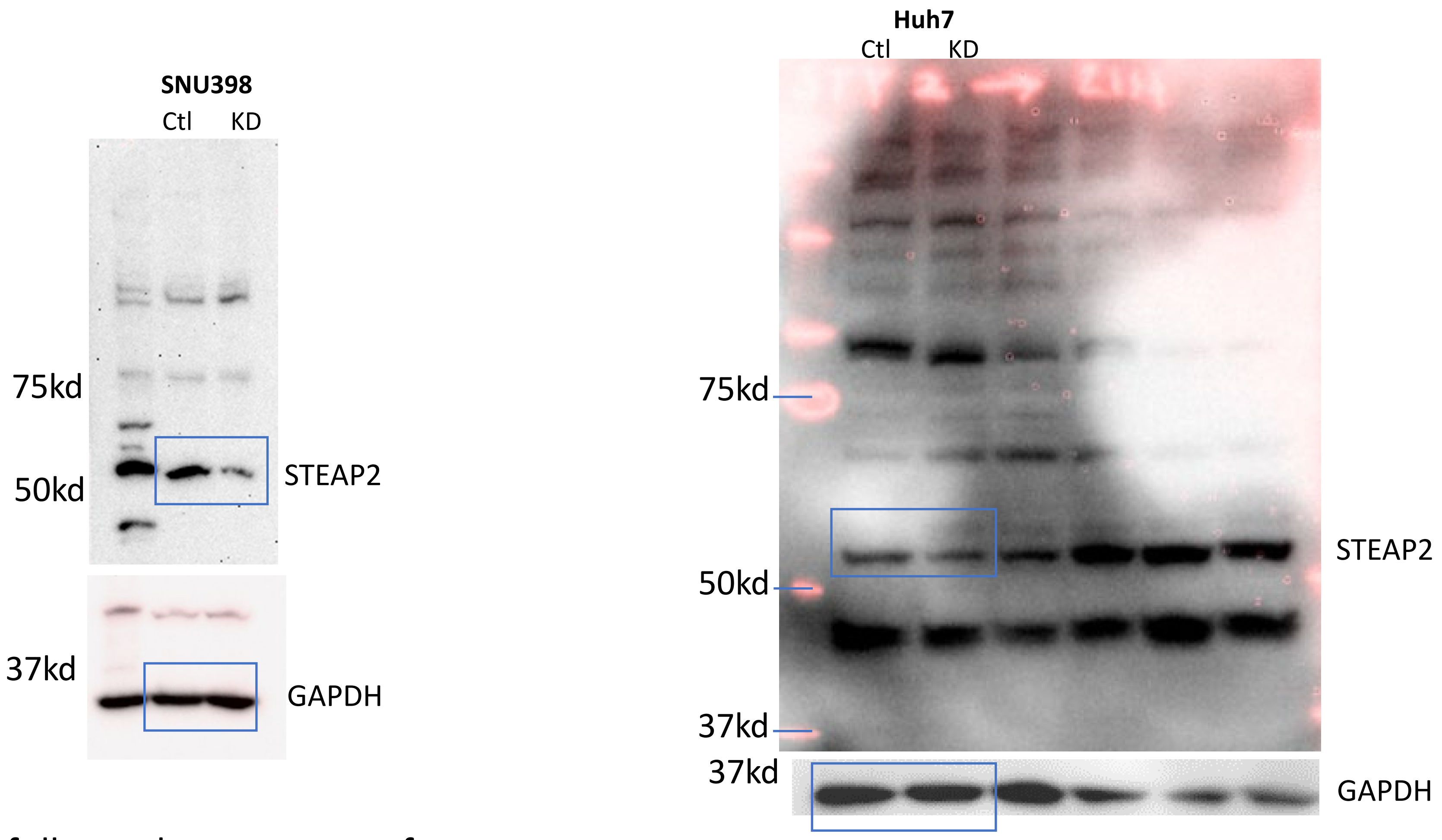

The full membrane was cut for probing STEAP2 and GAPDH separately.

STEAP2 was first probed in the full membrane. The membrane below 50kd marker was then cut and probed for GAPDH.

Fig 4C & D

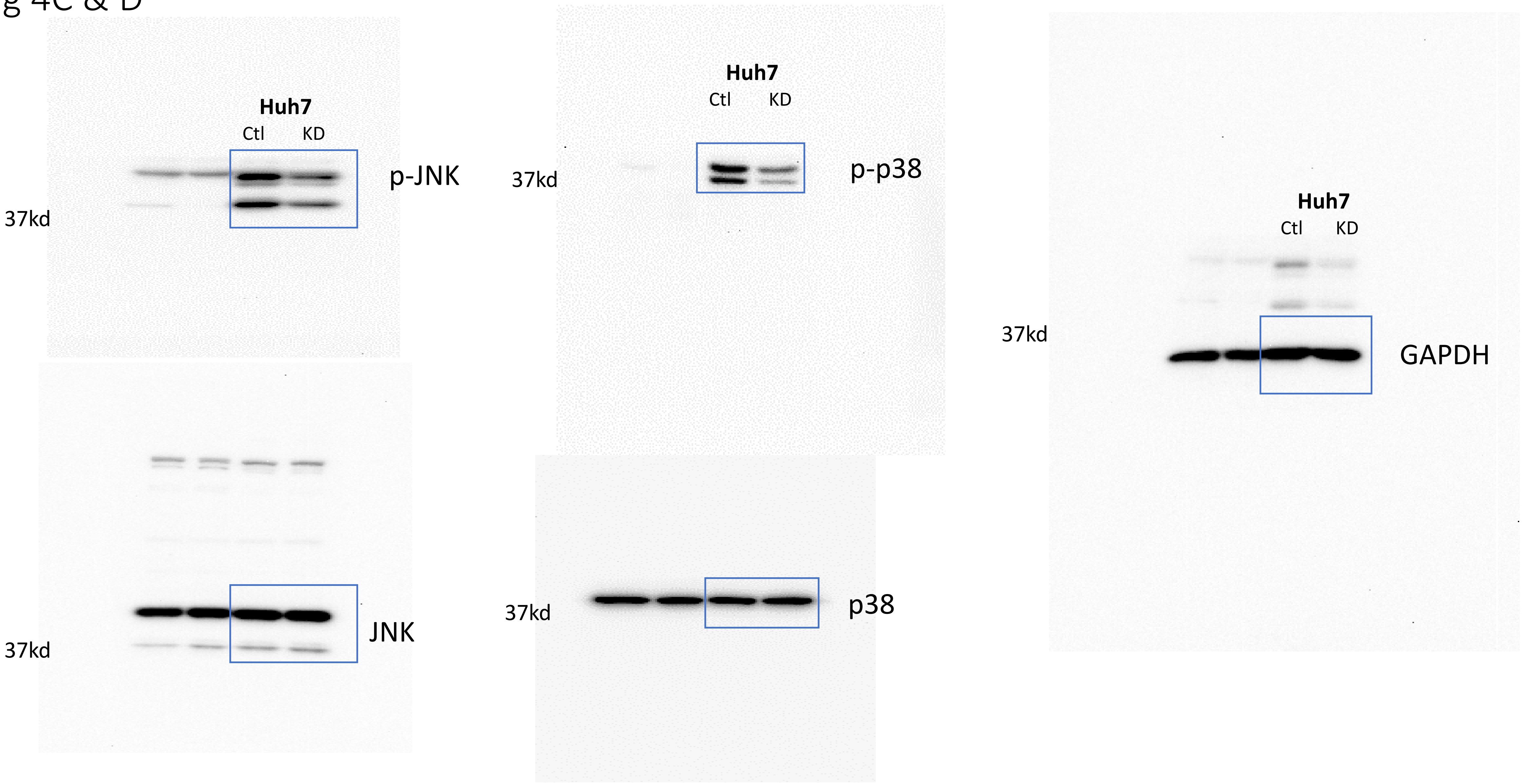

The membranes were cut above and below 37 kd marker before probing for the indicated proteins.

Fig 4C &D

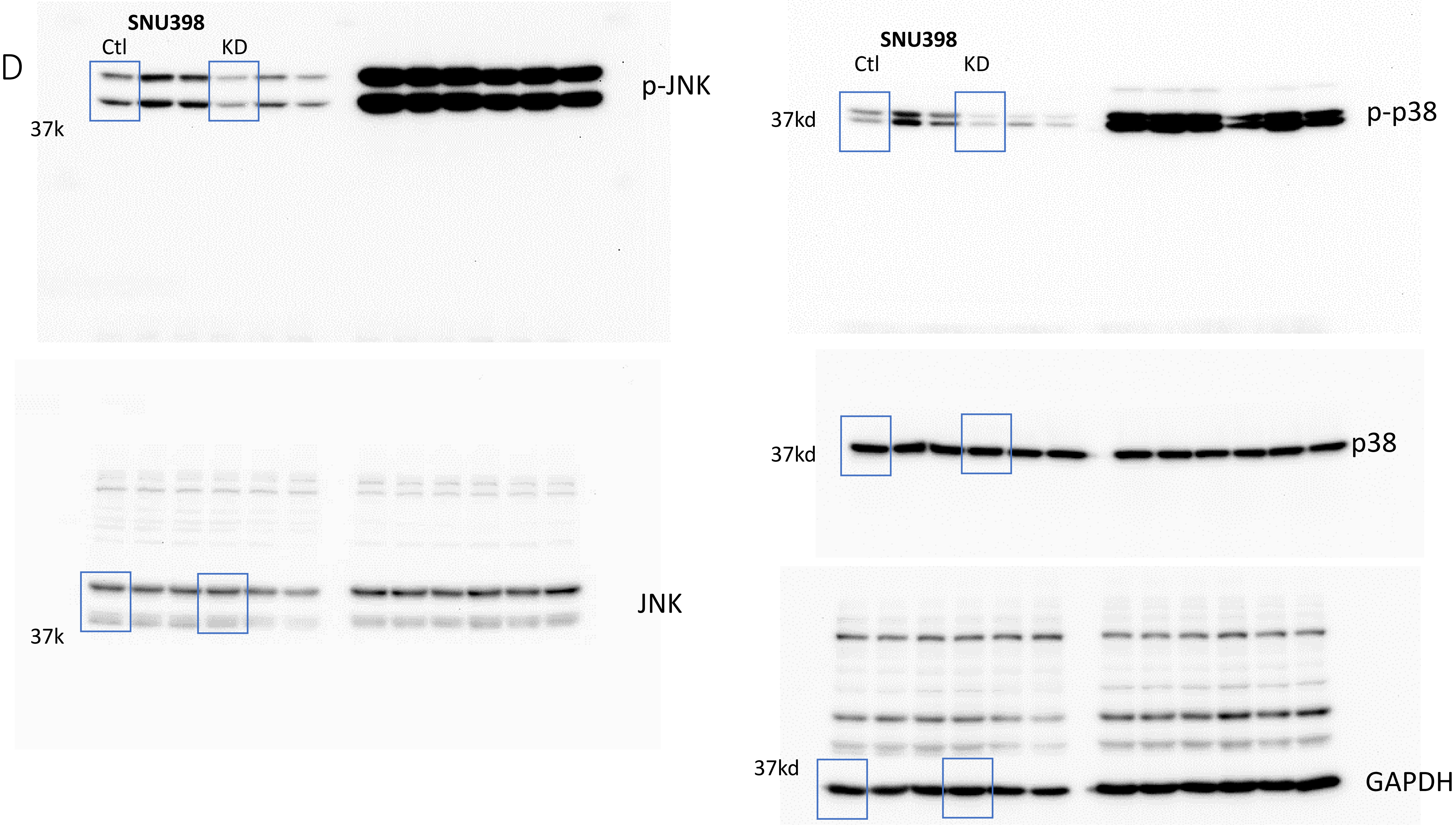

The membranes were cut above and below 37 kd marker before probing for the indicated proteins.

Fig 5A & B

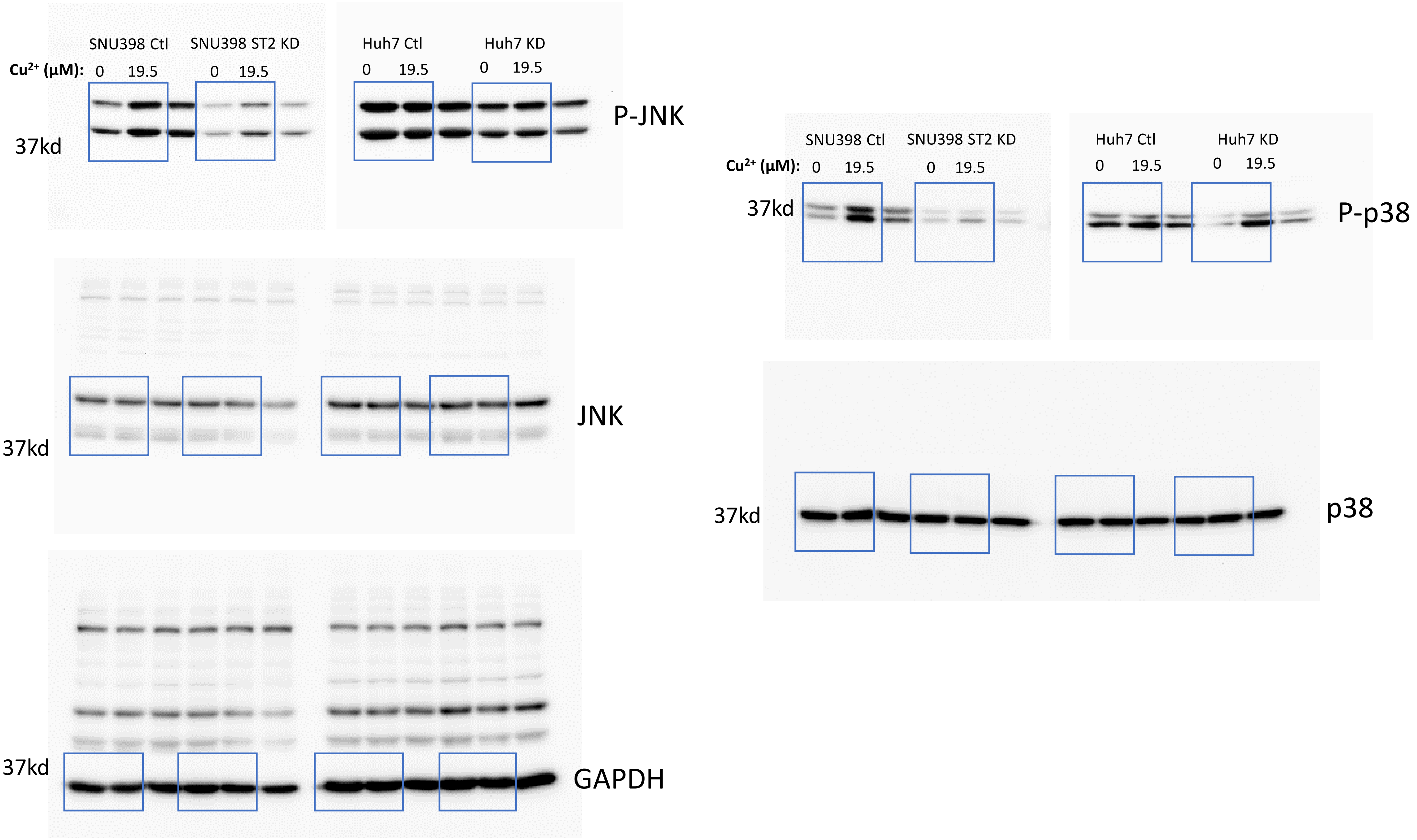

The membranes were cut above and below 37 kd marker before probing for the indicated proteins.

Fig 6A

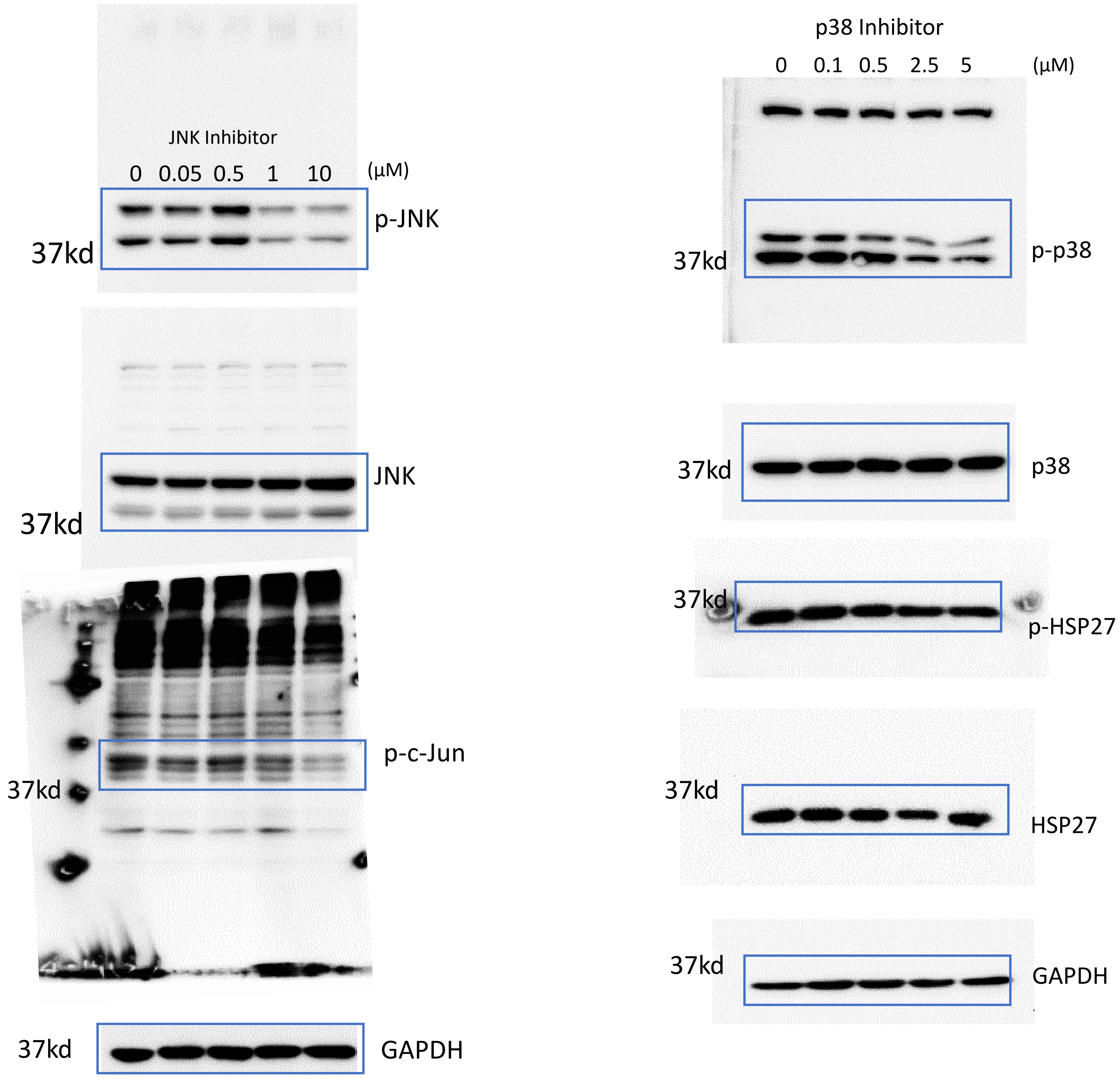

The membranes were cut above and below 37 kd marker before probing for the indicated proteins.

Fig 6c

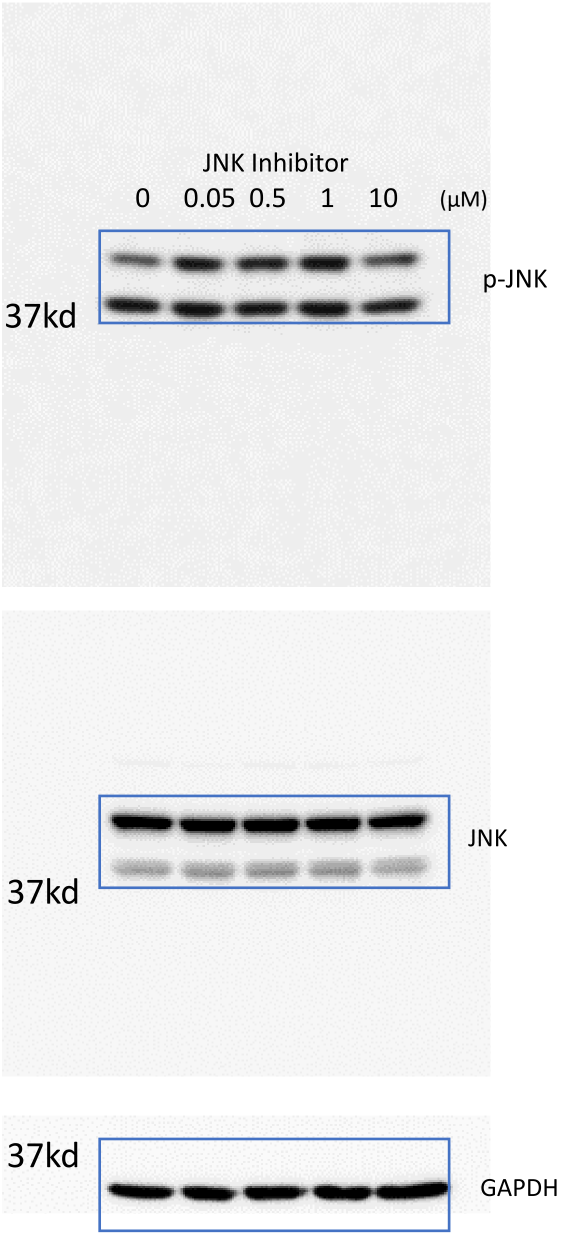

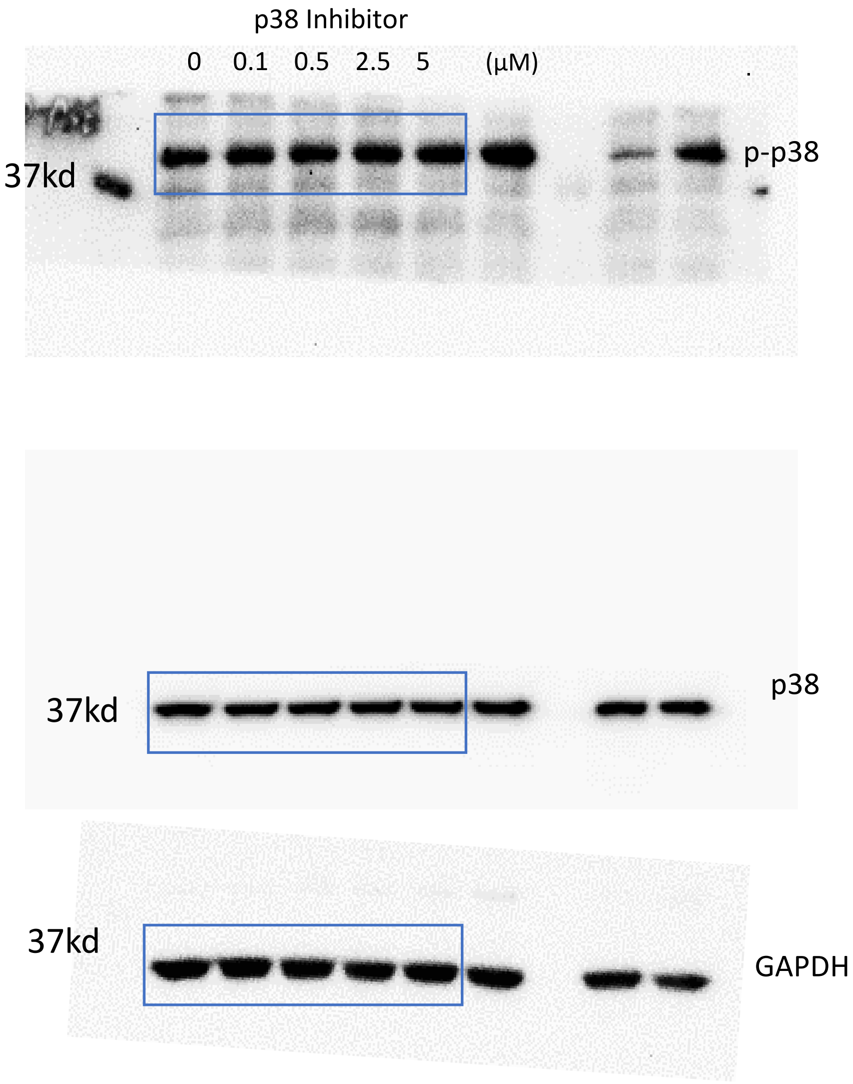

The membranes were cut above and below 37 kd marker before probing for the indicated proteins.

Supplementary Fig 3

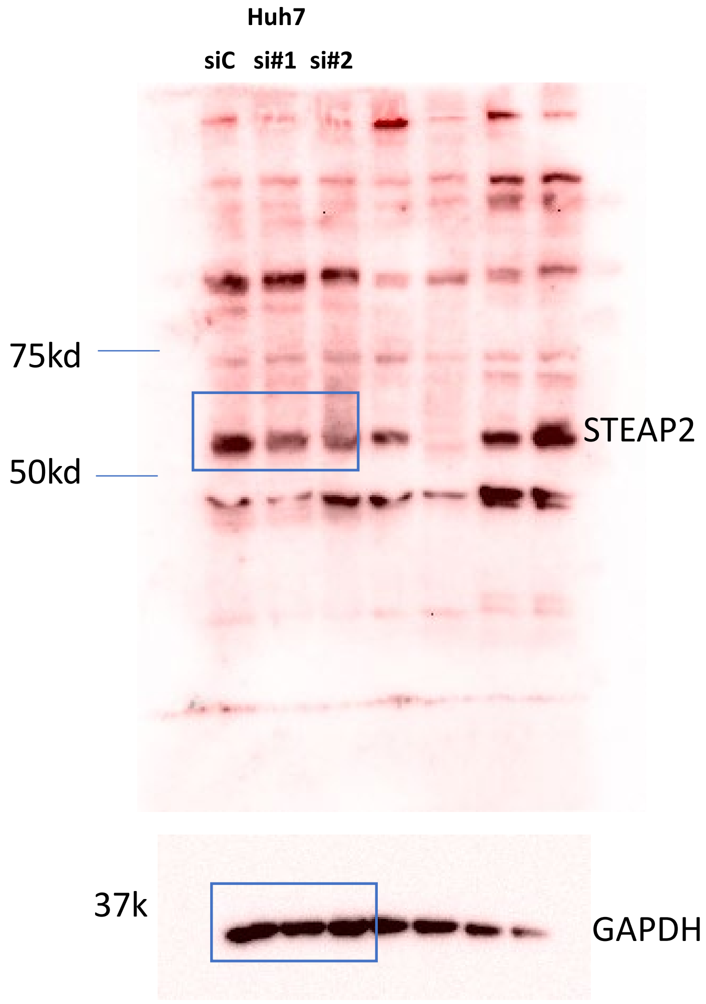

STEAP2 was first probed in the full membrane. The membrane below 50kd marker was then cut and probed for GAPDH.

Supplementary Fig 4

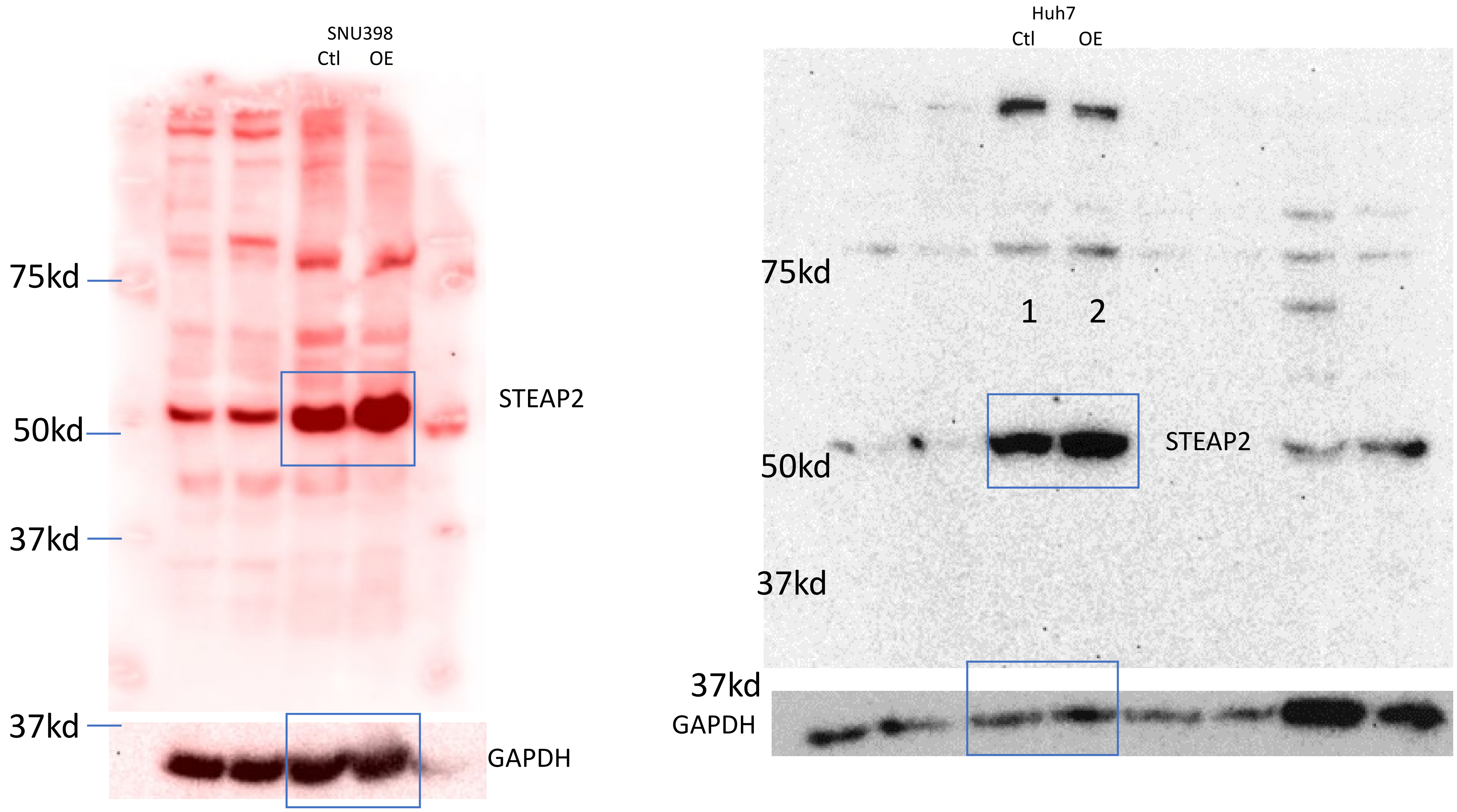

STEAP2 was first probed in the full membrane. The membrane below 50kd marker was then cut and probed for GAPDH.

Supplementary Fig 5A and 5B

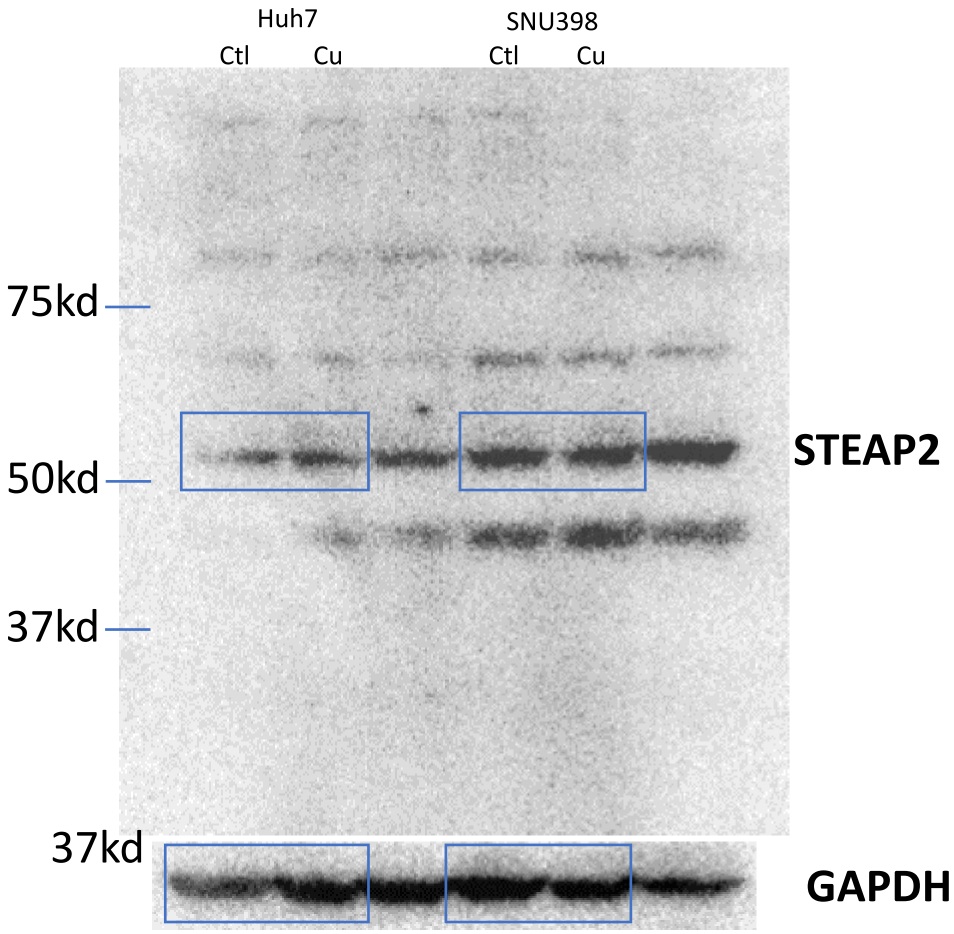

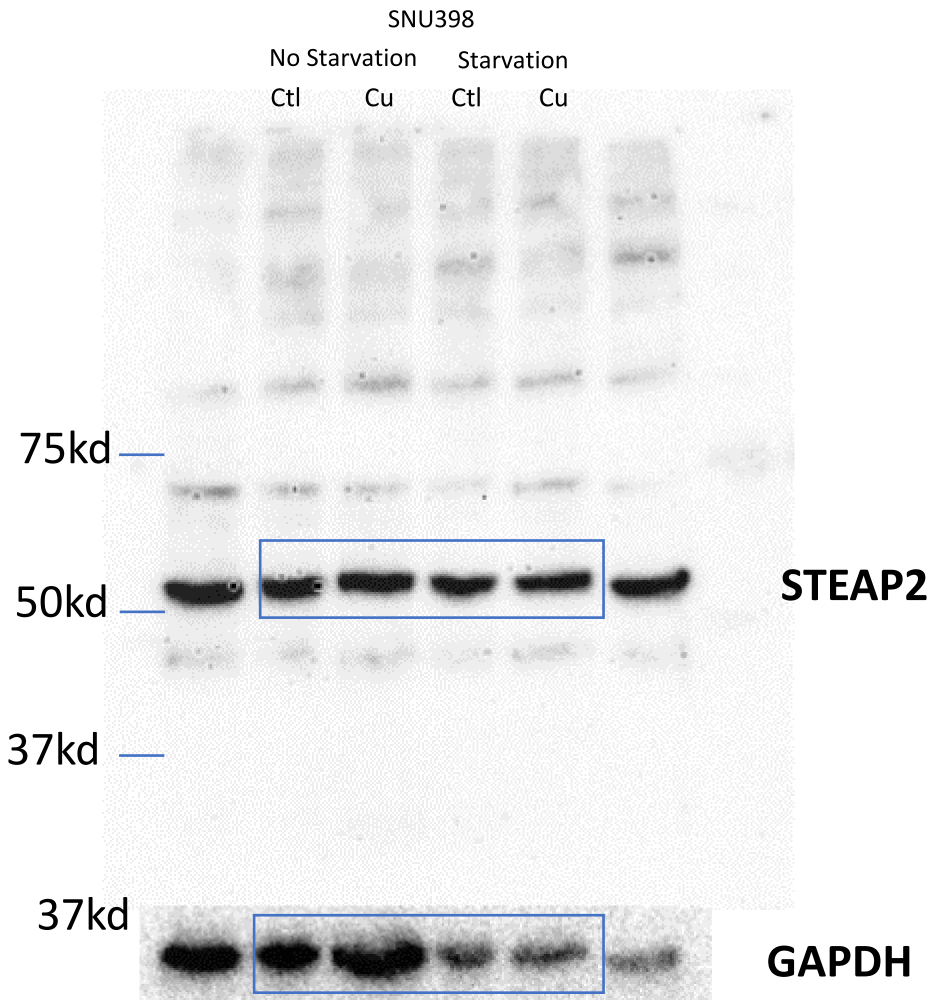

STEAP2 was first probed in the full membrane. The membrane below 50kd marker was then cut and probed for GAPDH.

Supplementary Fig 5C and 5D

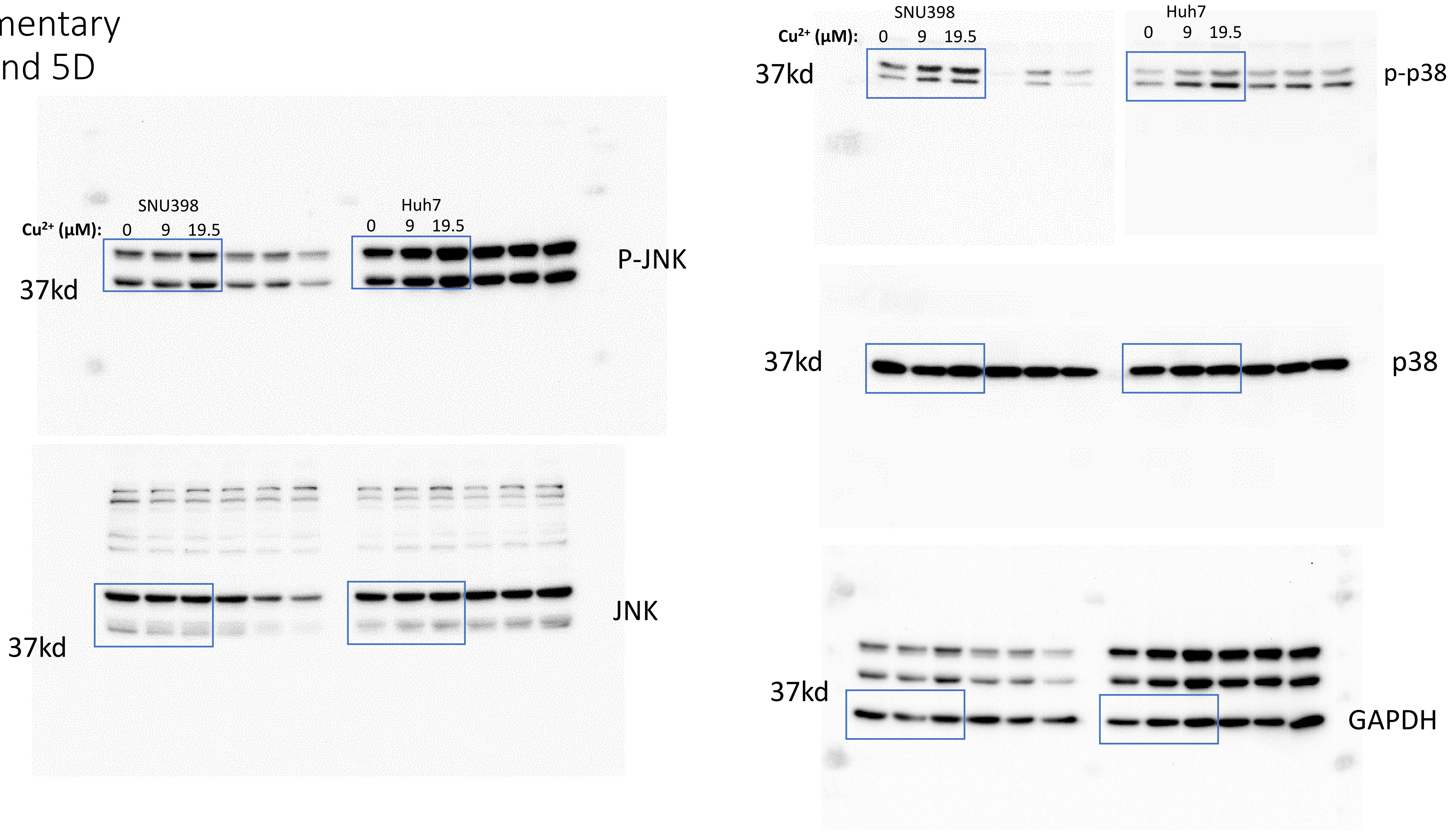

The membranes were cut above and below 37 kd marker before probing for the indicated proteins.
