# Supplementary material for: STEAP2 promotes hepatocellular carcinoma progression via increased copper levels and stress-activated MAP kinase activity
Source: Sci Rep. 2024 Jun 3;14:12753. doi: 10.1038/s41598-024-63368-2 (PMC11148201; doi:10.1038/s41598-024-63368-2)
Supplement: Supplementary file 2 — Supplementary Information 2. [file 41598_2024_63368_MOESM2_ESM.docx]

STEAP2 Promotes Hepatocellular Carcinoma Progression via Increased Copper Levels and Stress-Activated MAP Kinase Activity

**Carla Zeballos Torrez^1^, Acarizia Easley^1^, Hakim Bouamar^1^, Guixi Zheng^1^, Xiang Gu^1^, Junhua Yang^1^, Yu-Chiao Chiu^2^, Yidong Chen^2,3^, Glenn A. Halff^4^, Francisco G. Cigarroa^4*^, Lu-Zhe Sun^1*^**

^1^Department of Cell Systems & Anatomy, University of Texas Health Science Center at San Antonio, TX

^2^Department of Population Health Sciences, University of Texas Health Science Center at San Antonio, TX

^3^Greehey Children’s Cancer Research Institute, University of Texas Health Science Center at San Antonio, Texas, USA

^4^Transplant Center, University of Texas Health Science Center at San Antonio, TX

^*^Corresponding authors: Francisco G. Cigarroa, email: [Cigarroa@uthscsa.edu](mailto:Cigarroa@uthscsa.edu). Lu-Zhe Sun, email: [Sunl@uthscsa.edu](mailto:Sunl@uthscsa.edu)

**FUNDS:**

This study was in part supported by grants from Clayton Foundation, NCI T32CA148724, NCI F32CA228435, and NCI R01CA 247379.

**CONFLICT OF INTEREST**

The authors declare no conflict of interest.

List of abbreviations: Fetal bovine serum (FBS), six Transmembrane Epithelial Antigen of Prostate 2 (STEAP2), hepatocellular carcinoma (HCC), knockdown (KD), overexpression (OE), Tris buffered saline with Tween (TBST), Visualization and Integrated Discovery (DAVID), The Cancer Genome Atlas (TCGA), tumor necrosis factor-related apoptosis inducing ligand (TRAIL)

**Supplementary Data File**

**Supplement Table 1:** **Patient Demographics of Tissue Collected for RNA Sequencing**

| Sample ID | Grade | Hepatitis C | Cirrhosis | Diabetes | BMI > 25 |
| --- | --- | --- | --- | --- | --- |
| 1 | Grade 4 | No | Yes | Yes | Yes |
| 12 | Grade 2 | No | NA | Yes | Yes |
| 13 | Grade 2/3 | No | No | No | No |
| 15 | Grade 3 | Yes | Yes | No | Yes |
| 19 | Mod Diff | Yes | Yes | Yes | Yes |
| 34 | Grade 2 | Yes | NA* | NA* | NA* |
| 36 | Grade 2 | Yes | NA* | NA* | NA* |
| 37 | Grade 2 | Yes | NA* | NA* | NA* |
| 77 | Grade 2/3 | NA* | Yes | Yes | Yes |

*Data not available (NA)

**Supplement Table 2: Demographics of Additional Hispanic Patient Samples Collected for Cooper Analysis and IHC**

| Sample ID | Grade | Hepatitis C | Cirrhosis | Diabetes | BMI > 25 |
| --- | --- | --- | --- | --- | --- |
| 2 | Grade 2 | Yes | Yes | No | Yes |
| 4 | Grade 4 | Yes | No | Yes | Yes |
| 6 | Grade 3-4 | No | Yes | Yes | Yes |
| 20 | Grade 4 | Yes | No | No | Yes |
| 21 | Grade 2 | No | No | Yes | Yes |
| 81 | Grade 4 | Yes | Yes | No | Yes |

**Supplement Table 3: Demographics of Caucasian Patient Samples Collected for RT-PCR**

| Sample ID | Grade | Hepatitis C | Cirrhosis | Diabetes | BMI > 25 |
| --- | --- | --- | --- | --- | --- |
| 9 | Grade 4 | Yes | Yes | Yes | Yes |
| 45 | Grade 3 | Yes | Yes | No | No |
| 46 | Mod Diff | Yes | No | No | Yes |
| 47 | Grade 2 | No | No | Yes | Yes |
| 48 | Grade 2 | Yes | Yes | No | Yes |
| 50 | Grade2 | No | No | No | Yes |

**Supplement Table 4: List of Genes Enriched in Control Cells from Anastassiou Multicancer Invasiveness Signature**

|  | **Probe** | **Rank in Gene List** | **Log_2_FC (Ctl/STEAP2 KD)** | **Core Enrichment** |
| --- | --- | --- | --- | --- |
| 1 | SULF1 | 17 | 2.431 | Yes |
| 2 | COMP | 107 | 1.692 | Yes |
| 3 | COL5A1 | 119 | 1.656 | Yes |
| 4 | PLAU | 127 | 1.615 | Yes |
| 5 | LOXL2 | 143 | 1.55 | Yes |
| 6 | COL1A1 | 357 | 1.064 | Yes |
| 7 | COL5A2 | 410 | 1.004 | Yes |
| 8 | DCN | 554 | 0.861 | Yes |
| 9 | GREM1 | 576 | 0.838 | Yes |
| 10 | LUM | 622 | 0.811 | Yes |
| 11 | MMP2 | 627 | 0.808 | Yes |
| 12 | COL3A1 | 786 | 0.71 | Yes |
| 13 | RCN3 | 885 | 0.667 | Yes |
| 14 | THY1 | 1046 | 0.606 | Yes |
| 15 | LOX | 1089 | 0.59 | Yes |
| 16 | LGALS1 | 1144 | 0.571 | Yes |
| 17 | SERPINF1 | 1208 | 0.553 | Yes |
| 18 | SPARC | 1246 | 0.54 | Yes |
| 19 | COL11A1 | 1629 | 0.462 | Yes |
| 20 | EPYC | 1643 | 0.46 | Yes |
| 21 | EDNRA | 1928 | 0.414 | No |
| 22 | MMP11 | 2280 | 0.366 | No |
| 23 | FAP | 2467 | 0.343 | No |
| 24 | GLT8D2 | 3252 | 0.265 | No |
| 25 | POSTN | 3258 | 0.265 | No |
| 26 | ITGBL1 | 5100 | 0.121 | No |
| 27 | CRISPLD2 | 5761 | 0.075 | No |
| 28 | RAB31 | 5835 | 0.07 | No |
| 29 | COL6A2 | 5976 | 0.06 | No |
| 30 | COL10A1 | 6195 | 0.044 | No |
| 31 | SPOCK1 | 7588 | -0.045 | No |
| 32 | C7orf10 | 7924 | -0.068 | No |
| 33 | C1QTNF3 | 8192 | -0.088 | No |
| 34 | OLFML2B | 8262 | -0.093 | No |
| 35 | CTSK | 8368 | -0.1 | No |
| 36 | FBN1 | 10148 | -0.24 | No |
| 37 | TIMP3 | 10305 | -0.253 | No |
| 38 | VCAN | 11048 | -0.329 | No |
| 39 | FN1 | 11214 | -0.347 | No |
| 40 | NID2 | 11464 | -0.38 | No |
| 41 | NUAK1 | 11957 | -0.457 | No |
| 42 | SFRP4 | 12011 | -0.468 | No |
| 43 | TMEM158 | 12050 | -0.475 | No |
| 44 | PCOLCE | 12154 | -0.496 | No |
| 45 | COL6A3 | 12542 | -0.581 | No |
| 46 | PRRX1 | 13263 | -0.842 | No |
| 47 | COL1A2 | 13486 | -0.979 | No |
| 48 | ACTA2 | 13731 | -1.305 | No |
| 49 | INHBA | 13776 | -1.409 | No |


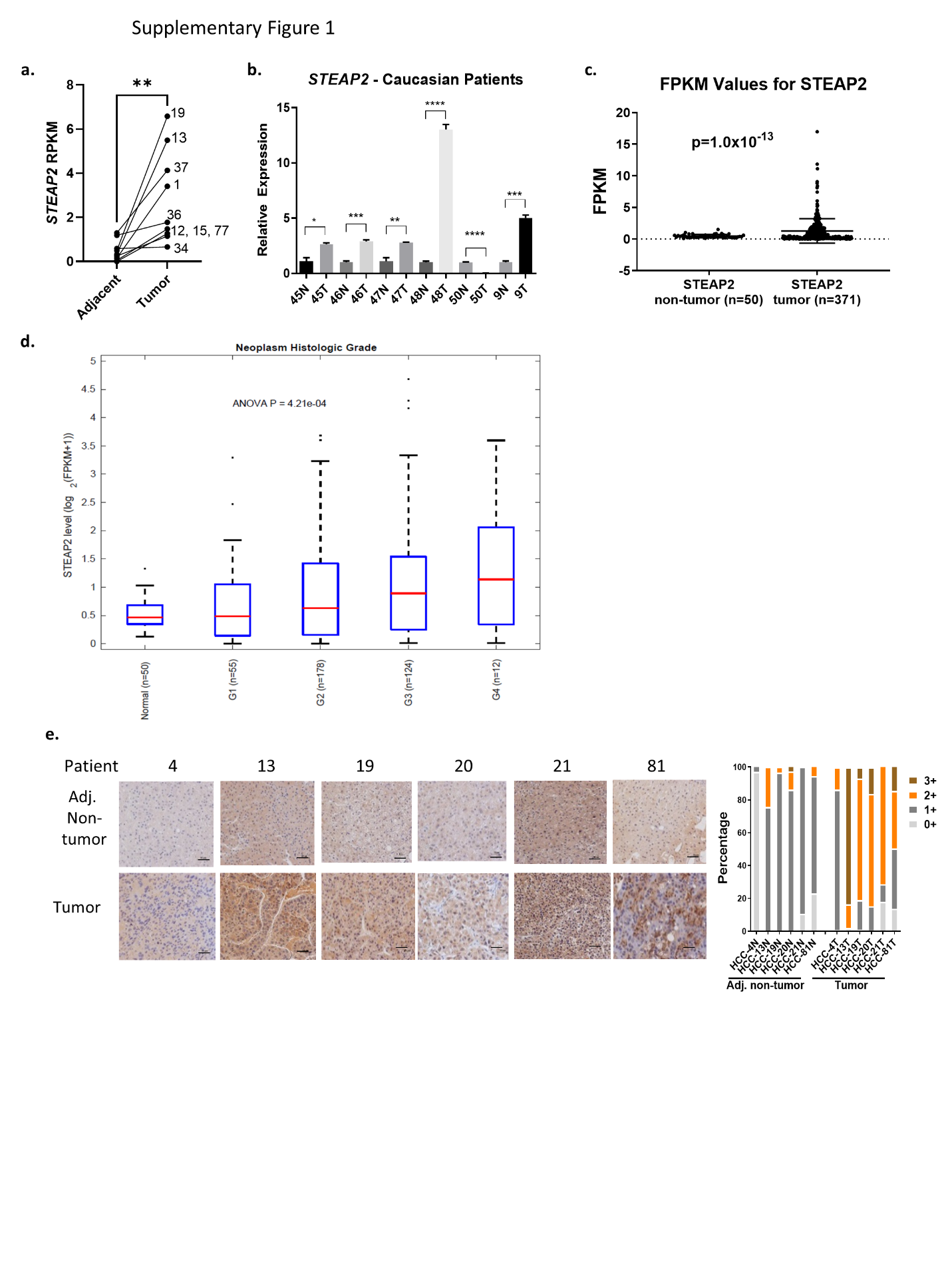


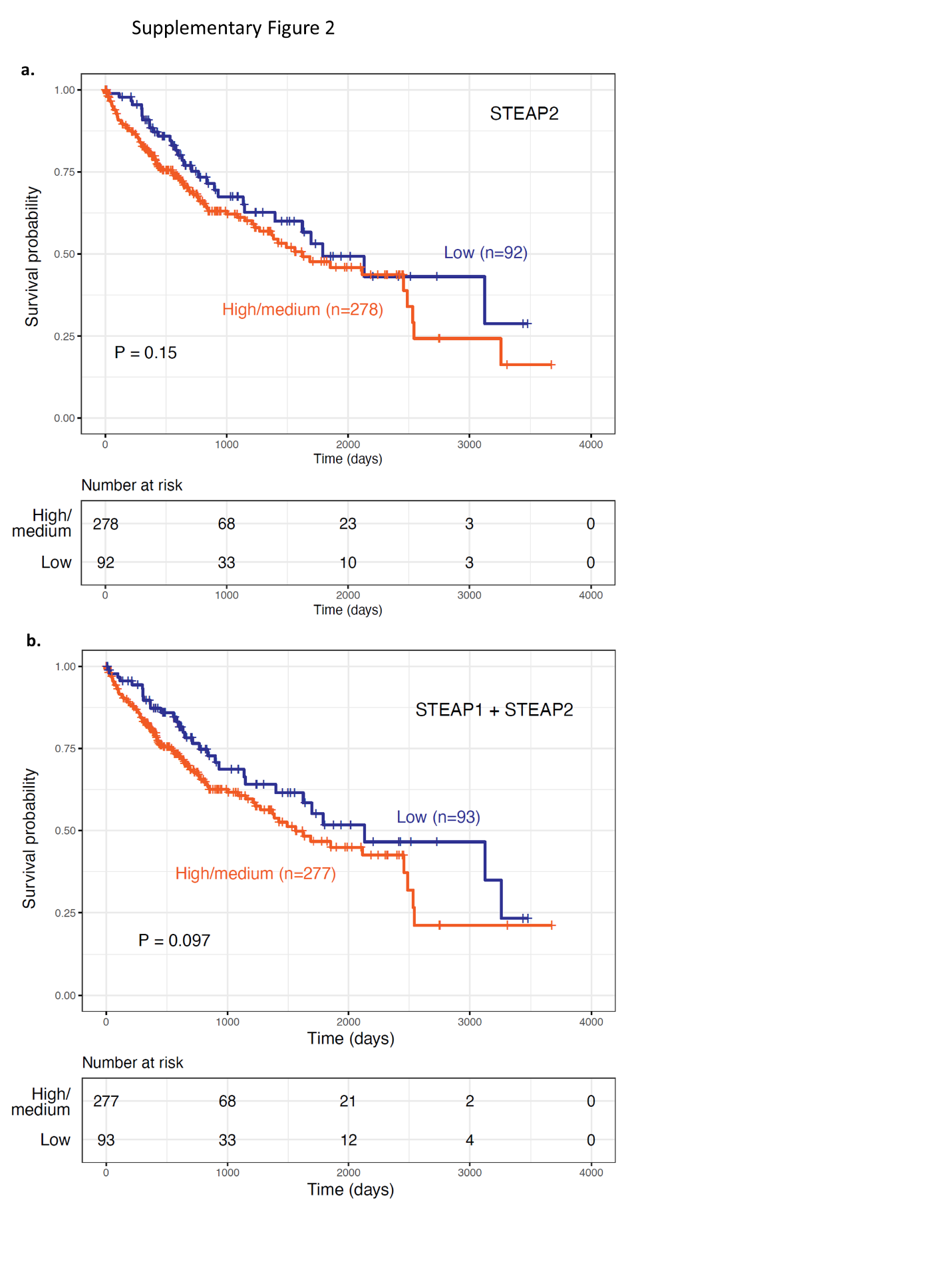


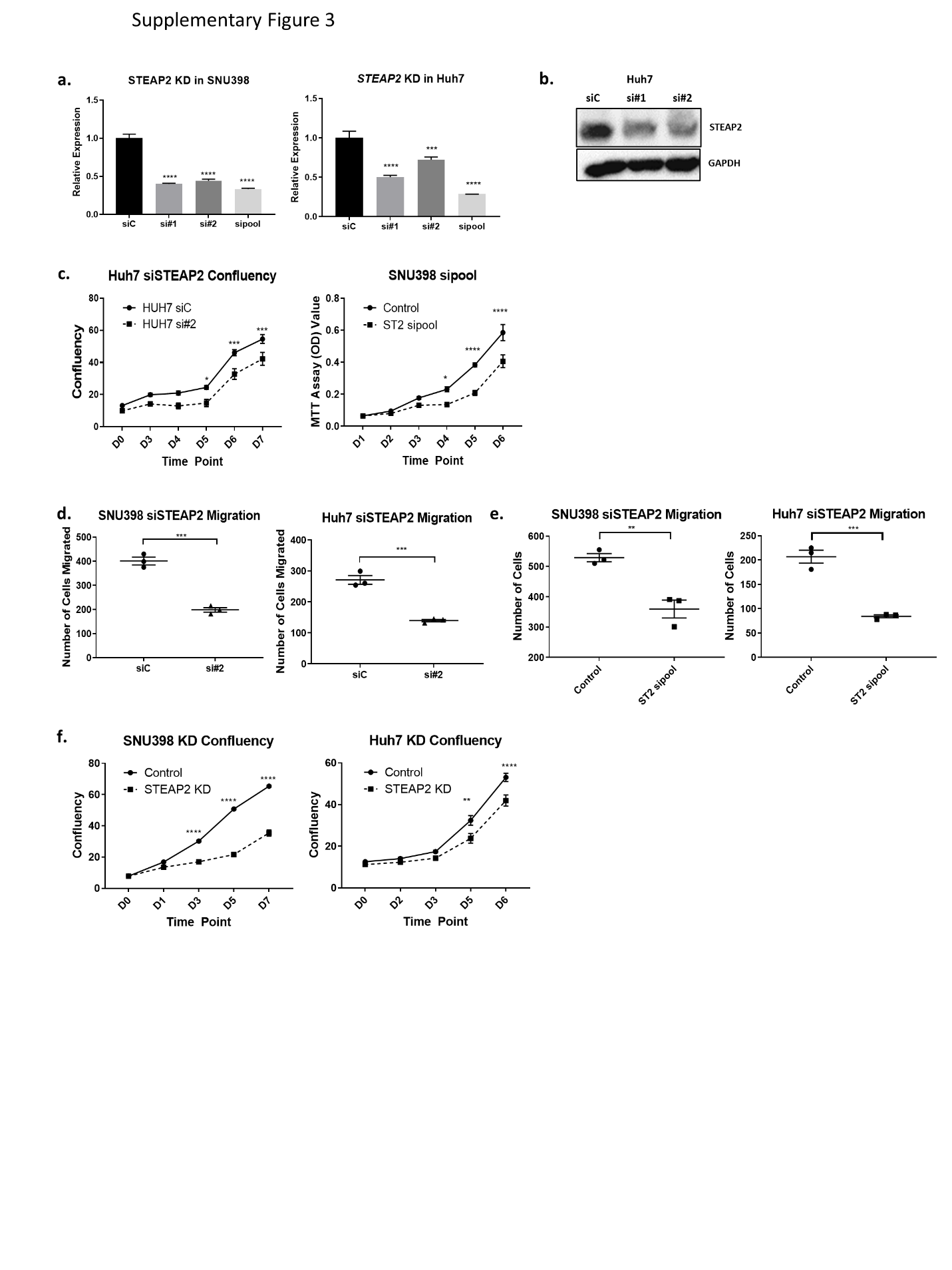


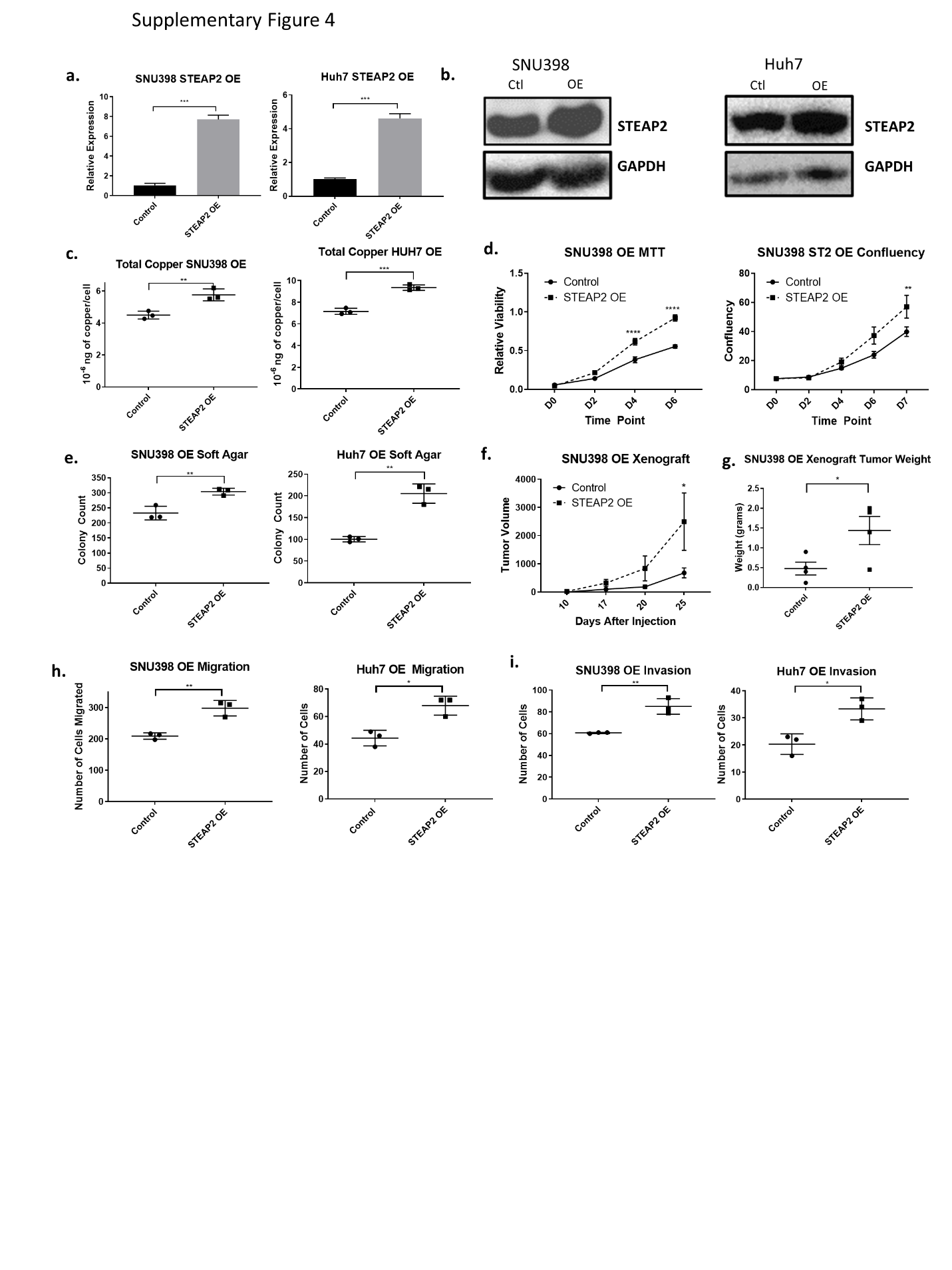


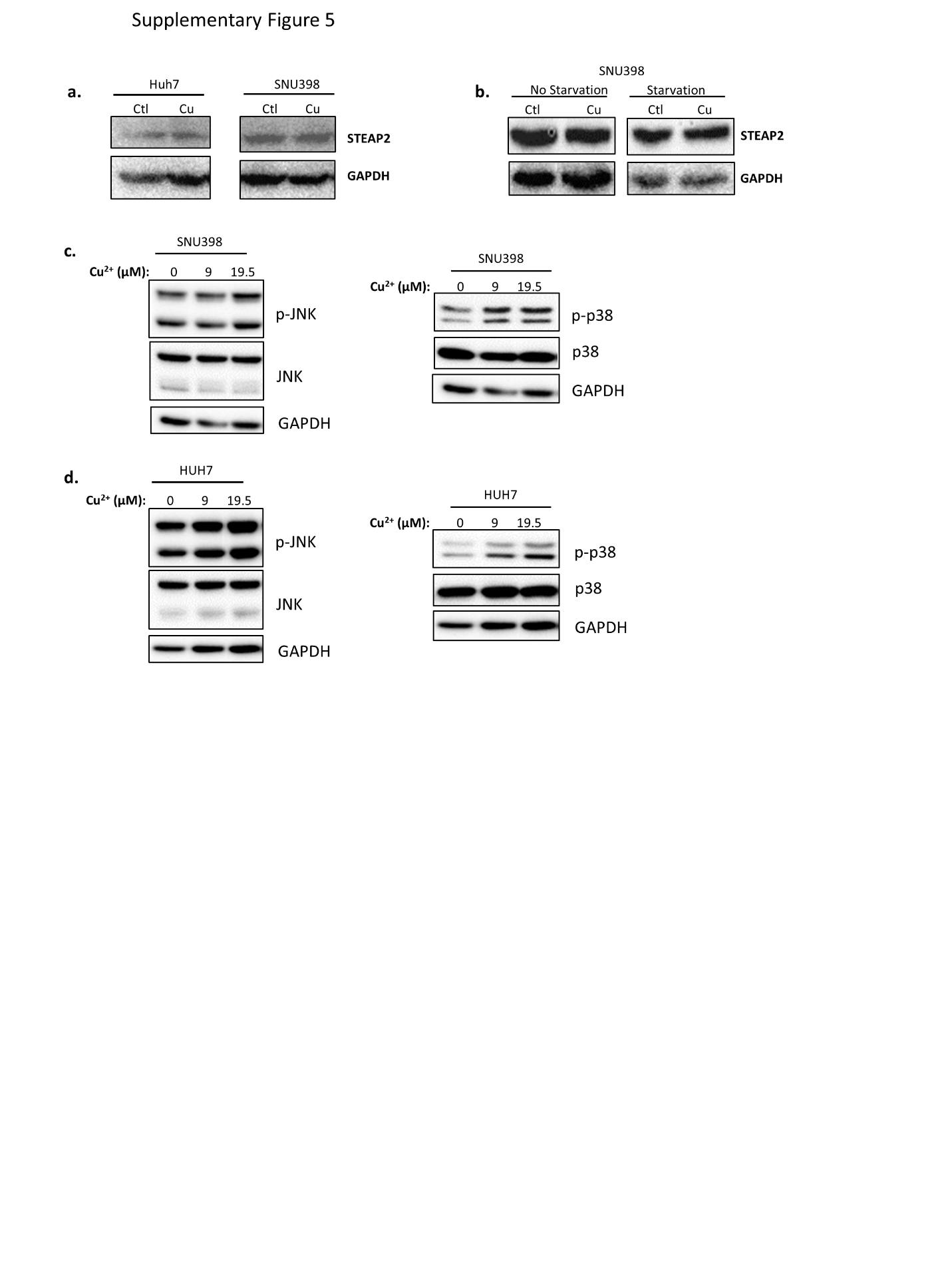


**Supplement Figures:**

**Supplement** **Figure 1: STEAP2 expression is increased in HCC. A.** *STEAP2* mRNA level expressed in reads per kilobase of transcript per million (RPKM) mapped reads in 9 paired Latino/Hispanic HCC tumor and adjacent non-tumor liver tissues **B.** *STEAP2* mRNA levels in HCC tumor (T) tissue compared with adjacent non-tumor (N) tissue from 6 Caucasian patients measured by real time RT-PCR. Tumor *STEAP2* mRNA level was normalized by its own adjacent non-tumor tissue STEAP2 mRNA level expressed as relative expression. **C.** *STEAP2* mRNA level expressed in Fragment per kilobase of transcript per million (FPKM) mapped reads in tumor tissue (n=371) compared to non-tumor tissue (n=50) in The Cancer Genome Atlas (TCGA) dataset. **D.** *STEAP2* expression levels increase with tumor grade in the TCGA dataset. **E.** IHC staining of STEAP2 in paired adj. non-tumor & HCC tumor tissues. Scale bar: 50 µm. The plot shows the average percent area of 3-4 representative high-power fields in each tissue section with different staining intensity from low (0+) to high (3+) analyzed with Leica Aperio Digital ImageScope. The ***P*-value** is 1.37e-17 comparing non-tumor with tumor groups using 2-way repeated-measure ANOVA after merging the percentage values weighted by the staining intensity score for each patient.

**Supplement Figure 2: Prognostic relevance of *STEAP2* and *STEAP1* to overall survival in the TCGA-LIHC cohort. A.** Patients were divided into high/medium and low expression groups based on the 25^th^ percentile of *STEAP2* expression. Statistical significance was assessed by the logrank test. The number of patients at risk at each time point is indicated. **B.** Z-scores were calculated for *STEAP2* and *STEAP1* in individual patients against the entire cohort. The mean of *STEAP2* and *STEAP1* z-scores was used to group patients, and the 25^th^ percentile was used as the cutoff. Statistical significance was assessed by the logrank test.

**Supplement Figure 3: STEAP2 knockdown inhibits growth *in vitro* and migration*.* A.** Confirmation of STEAP2 mRNA knockdown by transient transfection of STEAP2 siRNAs in HCC cell lines with qRT-PCR. GAPDH transcript was used for normalization. siSTEAP2 #1 targets the open reading frame (ORF), siSTEAP2 #2 targets the 3’ untranslated region, siSTEAP2 pool includes siSTEAP2 #1 and #2 and two additional siSTEAP2 that target the ORF. **B.** Confirmation of STEAP2 knockdown in HCC cell lines via Western Blot. GAPDH protein level was used to validate equal sample loading. Western blot image was cropped to show STEAP2 protein band. **C.** STEAP2 knockdown with siRNA inhibited HCC cell growth in confluency assay (left) and MTT assay (right). **D.** STEAP2 knockdown with siRNA inhibited HCC cell migration in transwell assay for siSTEAP2 #2 and for siSTEAP2 pool (**E.**). **F.** Stable STEAP2 Knockdown decreases cell growth via confluency assay. **P*<0.05; ***P*<0.01; ****P*<0.001. *****P*<0.0001 with unpaired T-test or two-way ANOVA and Sidak test.

**Supplementary** **Figure 4: STEAP2 overexpression increases copper levels, promotes growth *in vitro* and *in vivo*, and increases migration.** *STEAP2* cDNA plasmid and matched control plasmid, pCDH-CMV-MCS-EF1-coGFP, were transfected into SNU398 and Huh7 cells via lentiviral infection. **A.** Confirmation of *STEAP2* overexpression (OE) in HCC cell lines in its transcript level with qRT-PCR. GAPDH transcript was used for normalization. **B.** STEAP2 OE was confirmed at the protein level with Western blotting. GAPDH protein level was used to validate equal sample loading. Western blot image was cropped to show STEAP2 protein band. **C.** Copper levels increased in STEAP2 OE cells; measured via ICP-MS. STEAP2 OE increased HCC cell growth in MTT assay for SNU398 cells (**D.**), anchorage independent growth assay for SNU398 and Huh7 (**E.**). **F.** Growth curve of tumors formed by STEAP2 OE and matched Control SNU398 cells in male nude mice. Tumor volume was calculated using the formula: v=length x width^2^ x 0.5. Each data point represents the mean±SEM of four tumors. **G.** Tumors excised from euthanized mice were weighed at the end of the experiment. Each data point represents a tumor with Mean±SEM also presented. STEAP2 OE increased HCC cell migration (**H.**) and invasion (**I.**) in transwell assay. **P*<0.05; ***P*<0.01; ****P*<0.001. *****P*<0.0001 with unpaired T-test, two-way ANOVA and Sidak test.

**Supplementary** **Figure 5: Effect of copper supplementation on STEAP2 expression and phosphorylation of JNK and p38.** **A.** STEAP2 protein levels were unchanged with copper (19.5 µM) supplementation in culture medium for 24 hours in the HCC cells. **B.** SNU398 cells serum starved for 48 hours (on right), STEAP2 levels were unchanged with copper (19.5 µM) supplementation. **C.** Copper supplementation increases phosphorylation of JNK at T183/Y185 and phosphorylation of p38 at T180/Y185 in a dose dependent manner in parental SNU398 cells and Huh7 cells (**D.**). Western blot image was cropped to show appropriate protein band.

**Supplement Methods**:

**RNA extraction and sequencing:** Total RNA was isolated from cells and tissues using RNA Mini Spin Column of Enzy Max LLC (Lexington, KY) according to the manufacturer’s instructions. DNase (Thermo Scientific, Rockford, IL, Cat.# EN0521) was added to the first wash solution at 10µg/70µl and incubated for 15 min at room temperature to remove genomic DNA contamination. The quality of RNA samples was analyzed with a Bioanalyzer (Agilent 2100 Bioanalyzer, Agilent Technologies, Santa Clara, CA) by the Mays Cancer Center’s Next Generation Sequencing Shared Resource. Samples with RNA Integrity Number of 7 or greater were used for RNA sequencing library construction using TruSeq Stranded mRNA Library Prep kit according to manufacturer's protocol (Cat.# RS‑122‑2002; Illumina, Inc.). Eight paired samples were sequenced in the Illumina HiSeq 2000 and one paired samples in HiSeq® 3000 system (Illumina, Inc.) using a 100bp paired-end sequencing protocol. All sequence reads were aligned to the UCSC human genome build hg19 using TopHat2, and bam files from the alignment were further processed using HTSeq-count [1] to obtain the read counts per gene in all samples. Sequencing coverage and quality statistics, and data analysis for differential expression genes were reported previously [2]. Expression abundance of each gene/isoform was converted in unit of read counts and fragment per kilobase of transcript per million transcripts mapped (FPKM), along with its distribution which will be examined to eliminate very low expressed transcripts (< 1 FPKM). The EdgeR algorithm [3] were used to estimate the differential expression in read counts and their statistical significance for the 9 tumor/non-tumor pairs. Significantly differentially expressed genes with FDR below 0.05 (or 5%) and fold-change greater than 2 were selected and manually inspected by using Integrative Genomic Viewer (IGV, <http://www.broadinstitute.org/software/igv/>). Functional assessment of these differentially expressed isoforms will be performed by using Database for Annotation, Visualization and Integrated Discovery (DAVID http://david.abcc.ncifcrf.gov/) [4].

**Copper Measurement**: Quantification of copper was accomplished using ICP-MS at the Northwestern University Quantitative Bio-element Imaging Center (QBIC). ICP-MS was performed on a computer-controlled (QTEGRA software) Thermo iCapQ ICP-MS (Thermo Fisher Scientific, Waltham, MA, USA) operating in KED mode and equipped with a ESI SC-2DX PrepFAST autosampler (Omaha, NE, USA). Tissue or cell samples were digested in concentrated trace nitric acid (> 69%, Thermo Fisher Scientific, Waltham, MA, USA) and hydrogen peroxide (> 30 %, for trace analysis, Sigma-Aldrich, St. Louis, MO, USA) and placed at 65 °C for at least 3 hours to allow for complete sample digestion. Ultra pure H_2_O (18.2 MΩ∙cm) was then added to produce a final solution of 5.0% nitric acid (v/v) in a total sample volume of 5 ml. Quantitative standards were made by the prepFAST system and used to generate calibration curves consisting of 100, 50, 20, 10, 5, 2, 1 ng/ml Cu. Each sample was acquired using 3 main (peak jumping) runs (100 sweeps). The isotopes selected for analysis were ^63^Cu and ^65^Cu (chosen as internal standards for data interpolation and machine stability). Instrument performance is optimized daily through autotuning followed by verification via a performance report (passing manufacturer specifications).

**Gene modulation**: For transient knockdown, a pool of STEAP2 siRNAs (Dharmacon) at 25 nM were transfected into the Huh-7 and SNU-398 HCC cell lines with Dharmafect 4 transfection reagent. The following siRNA sequences from Dharmacon ON-TARGETplus SMARTpool were used: siRNA J-010739-09 (CAACAAUAUUCAAGCGCGA), J-010739-10 (AGUCUUAAUCCUAUGCAAA), J-010739-11 (GGCCAGAUGAGCUAAAUUA), J-010739-12 (ACAAGUAUGCUGUCAAAUU). Individual siRNA #1 which targets the open reading frame J-010739-11 (GGCCAGAUGAGCUAAAUUA) and individual siRNA #2 which targets the 3’ untranslated region J-010739-10 (AGUCUUAAUCCUAUGCAAA) were used in separate experiments to confirm knockdown. For stable knockdown, control lentivector TRC2-pLKO.5-puro (Sigma Cat. #SHC201) and STEAP2 shRNA expressing lentivector (Sigma Cat. # TRCN0000294357) targeting GCCAGTGGTGGTAGCTATAAG were used. For ectopic expression of STEAP2, a full length STEAP2 cDNA with an HA tag sequence at C-terminus was provided by Cyagen (Santa Clara, California) in a plasmid, which was PCR-amplified and cloned into the lentivector pCDH-CMV-MCS-EF1-coGFP vector at XbaI and NheI sites. The sequence of the cloned STEAP2 cDNA was confirmed with DNA sequencing. These lentivectors were transfected into 293T cells for lentivirus production and the HCC cell lines were infected with the lentivirus for stable expression STEAP2 shRNA or cDNA as previously described[2].

**MTT and Soft Agar Assay**: Cells were plated in triplicate in a 96-well plate, and cell viability was determined by MTT 3-(4,5-dimethylthiazol-2-yl)-2,5-diphenyltetrazolium bromide (Sigma, Cat. # M2128) assay for 6 days. MTT at 2 mg/ml in PBS was added into each well and incubated for 2 hours at 37°C. DMSO was used to dissolve the formazan product, and the absorbance was measured at a 595 nm wavelength. To assay anchorage-independent cell growth in soft agar, HCC cells were suspended in medium with 0.4% agar, and plated on top of a bottom layer containing medium plus 0.8% agar. After two weeks, the colonies were stained with iodonitrotetrazolium chloride (Sigma, Cat. # I-8377) and counted.

**Migration and Invasion Assay**: The capacity of HCC cells to migrate was evaluated using 24-well Transwell inserts with a 8-μm pore size (Corning, Cat. # 353097). For migration assays, 5 x 10^4^ cells were added with serum-free RPMI 1640 in the upper chamber of the Transwell. The lower chamber contained RPMI with 10% FBS. For invasion assays, cells were seeded on Matrigel-coated Transwell inserts (BD Biosciences Pharmingen, Cat. #354480). After 18-hours of incubation at 37°C, the cells inside the insert were gently removed with a cotton swab. The migrated cells on the lower surface of the Transwell membrane were fixed, stained, and counted (five fields per insert in triplicate wells) using a light microscope.

**Immunoblot**: Protein was extracted from the tissue or cell pellets using Laemmli buffer with protease inhibitors. The concentration of proteins was quantified by the bicinchoninic acid protein assay (Thermo Scientific, Rockford, IL). Equal amount of total protein was applied to SDS-PAGE electrophoresis and then transferred to the nitrocellulose membrane under 100 V. The membrane was blocked with the Tris buffered saline with Tween (TBST) containing 5% milk and incubated with primary antibody overnight at 4°C. Washing steps of 10 minutes for 3 times were applied after primary antibody and appropriate secondary antibody incubation. Proteins were detected with the peroxidase-coupled secondary antibody from Sigma-Aldrich, using Electrochemiluminescence (ECL, Thermo Fisher Scientific, Grand Island, NY) and visualized using the FluorChem E imager with Alpha View software version 4.1.4 (Protein Simple, San Jose, CA). Antibodies used in the study included anti-phospho-p38(Thr180/Tyr182) (Cell Signaling, Cat. # 9211), anti-p38 (Cell Signaling, Cat. # 9212), anti-phospho-JNK(Thr183/Tyr185) (Cell Signaling, Cat. # 4671), anti-JNK (Cell Signaling, Cat. # 9252), anti-GAPDH (Calbiochem, Billerica, MA, Cat. # 80602-840), anti-Mouse IgG (Jackson immunoresearch, West Grove, PA, Cat. # 115-035-003) and anti-Rabbit IgG (Jackson immunoresearch, Cat. # 111-035-003). A rabbit polyclonal antibody to STEAP2 was custom-made by Twentyfirst Century Biochemicals, Inc. (Marlboro, MA) using the amino acid sequence of QQSDFYKIPIEIVNKT in an ectodomain of STEAP2 as immunogen. All Western blot membrane images were cropped to only show the indicated bands in the figures. Uncropped images are shown in the Original Western Blots file as supplementary materials. For detecting p38, p-p38, JNK, p-JNK, and GAPDH, Western blot membranes were often cut above and below 37-50 KD molecular marker before they were blotted with primary and secondary antibodies as we knew the expected bands were at or around 37-50 KD (see samples of full-length Western blots for these proteins in the supplementary “Original Western Blots” file) and wished to use a reduced size of membrane and thus a minimal amount of blotting solution to save the amount of antibodies in the blotting solution. Therefore, full length Western blot membrane images were not available for these Western blots.

**Immunohistochemistry**: IHC staining was performed on paraffin-embedded 4 µm tissue sections and mounted on poly-L-Lysine coated slides. Briefly, after deparaffinization in xylenes and rehydration through graded ethanol solutions, antigen retrieval was performed by submerging the sections into a sodium citrate solution (10 mM, pH 6.0) or a EDTA solution (1 mM, pH 8.0) at 95C for 15 to 30 minutes, in a microwave oven. The tissue sections were then treated with 3% hydrogen peroxide in methanol to suppress the endogenous peroxidase activity. Tissue sections were then incubated with an anti-STEAP2 antibody (Abcam, ab 174978) at 4C overnight. After washing, the sections were incubated with pre-diluted secondary antibody (BD Pharmingen), followed by further incubation with 3,3-diaminobenxidine tetrahydrochloride (DAB). Finally, the slides were counterstained with hematoxylin and mounted in an aqueous mounting medium. Appropriate positive and negative controls were stained in parallel. For negative controls, primary antibodies were replaced with PBS.

**References**

1. Anders, S., P.T. Pyl, and W. Huber, HTSeq--a Python framework to work with high-throughput sequencing data*.* *Bioinformatics*, 2015. **31**(2): p. 166-9.

2. Zheng, G., H. Bouamar, M. Cserhati, et al., Integrin alpha 6 is upregulated and drives hepatocellular carcinoma progression through integrin alpha6beta4 complex*.* *Int J Cancer*, 2022. **151**(6): p. 930-943.

3. Robinson, M.D., D.J. McCarthy, and G.K. Smyth, edgeR: a Bioconductor package for differential expression analysis of digital gene expression data*.* *Bioinformatics*, 2010. **26**(1): p. 139-40.

4. Huang da, W., B.T. Sherman, and R.A. Lempicki, Systematic and integrative analysis of large gene lists using DAVID bioinformatics resources*.* *Nat Protoc*, 2009. **4**(1): p. 44-57.
